# A Protein Inventory of Human Ribosome Biogenesis Reveals an Essential Function of Exportin 5 in 60S Subunit Export

**DOI:** 10.1371/journal.pbio.1000522

**Published:** 2010-10-26

**Authors:** Thomas Wild, Peter Horvath, Emanuel Wyler, Barbara Widmann, Lukas Badertscher, Ivo Zemp, Karol Kozak, Gabor Csucs, Elsebet Lund, Ulrike Kutay

**Affiliations:** 1 Institute of Biochemistry, ETH Zurich, Zurich, Switzerland; 2 Molecular Life Science Ph.D. Program, Zurich, Switzerland; 3 Light Microscopy Center, RISC, Department of Biology, ETH Zurich, Zurich, Switzerland; 4 University of Wisconsin, Madison, Wisconsin, United States of America; Albert Einstein College of Medicine

## Abstract

The assembly of ribosomal subunits in eukaryotes is a complex, multistep process so far mostly studied in yeast. In *S. cerevisiae*, more than 200 factors including ribosomal proteins and *trans*-acting factors are required for the ordered assembly of 40S and 60S ribosomal subunits. To date, only few human homologs of these yeast ribosome synthesis factors have been characterized. Here, we used a systematic RNA interference (RNAi) approach to analyze the contribution of 464 candidate factors to ribosomal subunit biogenesis in human cells. The screen was based on visual readouts, using inducible, fluorescent ribosomal proteins as reporters. By performing computer-based image analysis utilizing supervised machine-learning techniques, we obtained evidence for a functional link of 153 human proteins to ribosome synthesis. Our data show that core features of ribosome assembly are conserved from yeast to human, but differences exist for instance with respect to 60S subunit export. Unexpectedly, our RNAi screen uncovered a requirement for the export receptor Exportin 5 (Exp5) in nuclear export of 60S subunits in human cells. We show that Exp5, like the known 60S exportin Crm1, binds to pre-60S particles in a RanGTP-dependent manner. Interference with either Exp5 or Crm1 function blocks 60S export in both human cells and frog oocytes, whereas 40S export is compromised only upon inhibition of Crm1. Thus, 60S subunit export is dependent on at least two RanGTP-binding exportins in vertebrate cells.

## Abstract 2

A systematic search for human ribosome biogenesis factors shows conservation of many aspects of eukaryotic ribosome synthesis with the well-studied process in yeast and identifies an export route of 60S subunits that is specific for higher eukaryotes.

## Author Summary

Ribosomes are molecular machines that synthesize proteins and are found in every cell. Each ribosome has a small and a large subunit, each of which is in turn composed of ribosomal RNA and many ribosomal proteins. In eukaryotic cells, the generation of these ribosomal subunits is a complex process requiring the participation of hundreds of factors. Originating from the nucleolus (a nuclear region specialized in ribosome synthesis) immature ribosomal subunits pass through the nuclear interior and are exported to the cytoplasm, where their assembly is finalized. In recent years, it has become apparent that defects in ribosome production are associated with human diseases, but our knowledge about this fundamental process is largely based on knowledge derived from yeast, a unicellular eukaryote. We set out to systematically identify factors involved in making ribosomes in human cells by individually depleting around 500 different cellular proteins. Using microscopic analysis, we identified approximately 150 human ribosome synthesis factors, thereby significantly extending our knowledge about human ribosome biogenesis. Our dataset not only revealed many evolutionarily conserved aspects of this essential cellular process but also led us to characterize an export route for the large ribosomal subunit that is specific for higher eukaryotic cells.

## Introduction

The synthesis of ribosomal subunits is a major cellular task in all living organisms. Ribosomal subunit assembly requires deposition of numerous ribosomal proteins on ribosomal RNA (rRNA) to yield two ribonucleoprotein particles, a small and a large subunit, that together form a ribosome competent for protein translation. Our knowledge about ribosomal subunit biogenesis is most advanced for unicellular model organisms such as bacteria (*Escherichia coli*) and budding yeast (*S. cerevisiae*).

Pioneering early experiments demonstrated that simple subunits such as *E. coli* 30S [1] and 50S [2] can be reconstituted from rRNA and ribosomal proteins in vitro. Still, in vivo various non-ribosomal factors assist subunit assembly by promoting rRNA folding, modification, and protein deposition [3],[4]. With the increase in ribosome complexity and the establishment of cellular compartments in eukaryotes, ribosome biogenesis became dependent on a greater variety and number of *trans*-acting factors [5]–[7].

In yeast, more than 200 proteins are necessary for efficient ribosomal subunit assembly, which starts in the nucleolus with RNA polymerase I-mediated transcription of a large rRNA precursor molecule containing rRNA pieces of both subunits. Co-transcriptionally, the first *trans*-acting factors and ribosomal proteins assemble with the nascent pre-rRNA, generating an 80–90S precursor particle [8],[9]. During nuclear pre-rRNA maturation, numerous small nucleolar RNPs (snoRNPs) are involved in guiding and promoting various rRNA modification and processing reactions. The U3 snoRNP as part of the small subunit (SSU) processome is required for early endonucleolytic cleavage of the pre-rRNA at sites A0, A1, and A2 [6]. Cleavage at site A2 results in the conversion of the 90S pre-ribosome into separate pre-40S and pre-60S particles [10]. Subsequently, the two subunit precursors mature independently of each other through a series of rRNA processing and RNP remodeling steps in the nucleolus and nucleoplasm. After export from the nucleus, both subunits are subjected to final maturation in the cytoplasm, including the release of *trans*-acting factors and the completion of rRNA processing [11],[12].

Systematic approaches such as proteomic analysis of preribosomal particles and genetic screens have greatly helped the rapid advancement of our understanding of ribosome biogenesis in yeast and have yielded a comprehensive picture of the biogenesis pathway [7],[13]–[20]. In higher eukaryotes, this global view is still lacking, but particular aspects of ribosome biogenesis such as rRNA transcription and processing, snoRNP biogenesis and function, and nuclear export have been studied in different vertebrate model systems such as cultured cells and frog oocytes [6],[21]–[23]. These studies revealed that many aspects of ribosome synthesis are well conserved from yeast to human and many yeast *trans*-acting factors possess functional homologs in vertebrate species [12],[24]–[38]. However, for most human homologs of yeast *trans*-acting factors, a function in ribosome biogenesis has not yet been established. Moreover, vertebrate ribosome synthesis is dissimilar in several aspects, including the genomic organization of rDNA [39], differences in rRNA processing [6],[31],[40]–[43], and links to stress response pathways unique for higher eukaryotes [44].

Here we present a systematic approach to directly assess the functional requirement of 464 selected proteins in ribosome maturation in human cells. We depleted these factors by RNAi and scored for ribosome maturation defects using microscopic readouts. Our analysis provides a list of 153 proteins required for human ribosome synthesis. For 40S biogenesis, we can attribute their requirement to nucleolar, nucleoplasmic, or cytoplasmic maturation steps. Collectively, our data show that core features of 40S biogenesis are indeed conserved from yeast to humans. However, we also uncover unexpected differences such as the requirement of the pre-miRNA export receptor Exp5 for 60S subunit export to the cytoplasm.

## Results and Discussion

### Assays to Monitor Ribosomal Subunit Biogenesis in Human Cells

To identify proteins required for ribosome biogenesis by RNAi in HeLa cells, we established visual readouts suitable to detect defects in ribosomal subunit maturation in a high-throughput manner using microscopic endpoint assays.

**Figure 1:**
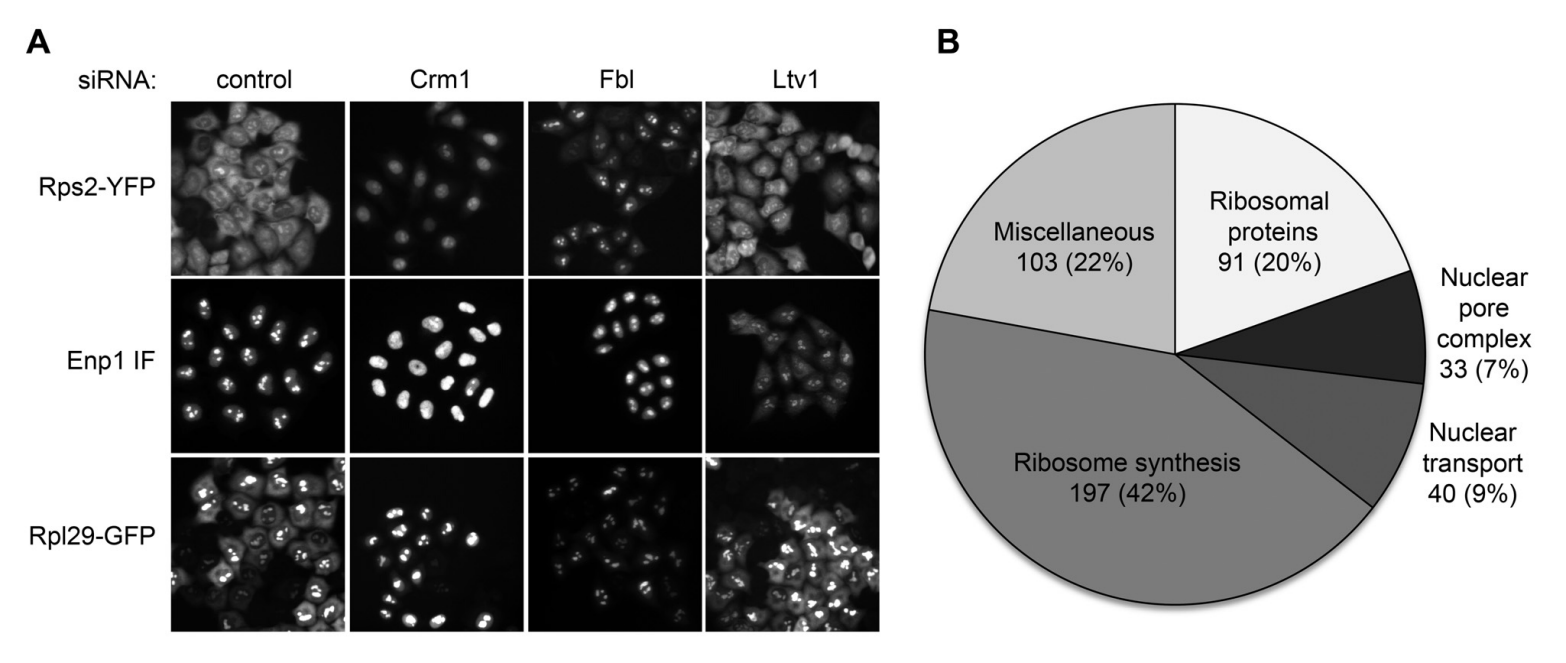
Readouts, phenotypes, and targets used for the systematic analysis of human ribosome biogenesis by RNAi. (A) Fluorescent images representing the different phenotypic categories used for detecting defects in 40S biogenesis (Rps2-YFP, Enp1 IF) and 60S biogenesis (Rpl29-GFP) after 3 d of RNAi in HeLa cells with the indicated siRNAs (10 nM). (B) Schematic representation of the siRNA target distribution to different protein categories. The list of all targets is given in Table S1.

For the 40S subunit, we applied two different readouts [45], namely an inducible, fluorescent ribosomal reporter protein, Rps2-YFP, and immunofluorescence (IF) analysis of the *trans*-acting factor Enp1(BYSL) [24]. Tetracycline-induced expression of Rps2-YFP allows for selective visualization of newly synthesized 40S subunits [45]. After 14 h of induction, Rps2-YFP-containing 40S have largely completed maturation, giving rise to a prominent cytoplasmic signal of the reporter (Figure 1A, control RNAi). RNAi against Crm1, the RanGTP-dependent nuclear export receptor required for both pre-40S and pre-60S export [34],[35], resulted in a nucleoplasmic accumulation of Rps2-YFP, reflecting the dependence of pre-40S export on Crm1. In contrast, inhibition of nucleolar steps of ribosome biogenesis, as exemplified by depletion of the box C/D snoRNPs-associated methyltransferase fibrillarin (Fbl) [10], led to accumulation of Rps2-YFP in nucleoli (Figure 1A). Thus, two phenotypes can be distinguished using the Rps2-YFP reporter, reflecting early (nucleolar) and late (nucleoplasmic) defects in nuclear pre-40S maturation.

The IF analysis of Enp1 complements the Rps2-YFP readout, as it allows for detection of both nuclear and cytoplasmic defects in 40S biogenesis. Enp1 is nucleolar at steady state but accompanies pre-40S to the cytoplasm from where it is recycled during cytoplasmic subunit maturation [45]. Inhibition of 40S export (Crm1 RNAi) resulted in a nucleoplasmic accumulation of Enp1, whereas interference with cytoplasmic 40S maturation led to cytoplasmic Enp1 localization, as observed upon downregulation of the 40S *trans*-acting factor Ltv1 (Figure 1A). Altogether, these readouts enable us to monitor the entire 40S maturation pathway, from the nucleoli through the nucleoplasm to the cytoplasm, and to identify factors required for subunit assembly and nuclear export as well as recycling of *trans*-acting factors in our RNAi screening approach.

To monitor 60S biogenesis, we established a HeLa cell line carrying an inducible copy of the ribosomal reporter protein Rpl29-GFP. Rpl29-GFP is efficiently incorporated into 60S subunits (Figure S1), and the GFP-readout faithfully illustrates the Crm1 dependency of 60S export (Figure 1A). Thus, this cell line is suitable to study nuclear 60S biogenesis, but currently we do not have the means to detect defects in cytoplasmic 60S maturation.

### Systematic RNAi Analysis of Candidate Factors for Human Ribosome Synthesis

Based on curation of the literature, we compiled a list of proteins with a known or potential function in human ribosome biogenesis (Figure 1B and Table S1), including ribosomal proteins, nuclear pore complex (NPC) components, proteins of the nucleo-cytoplasmic trafficking machinery, and potential or established human homologues of yeast *trans*-acting factors. Additionally, we included factors that function in various other cellular pathways that may or may not impact on ribosome biogenesis, like mRNA or tRNA metabolism, protein modification, and degradation (miscellaneous category).

The RNAi screen was performed in a 96-well format using three different siRNAs per target at an siRNA concentration of 10 nM. On each plate, three negative (Allstars siRNA, Qiagen) and three positive (Crm1 siRNA) controls were present. Image analysis was performed in an automated fashion, using image segmentation based on the identification of cell nuclei by Hoechst fluorescence as a first step. Then, 30 different features including the mean nuclear and cytoplasmic fluorescence intensities, morphological descriptors, and texture marks were extracted from the images using a customized version of Cell Profiler [46]. For example, nucleolar and nucleoplasmic accumulation of Rps2-YFP were distinguished by textural features. Phenotypic analysis of the dataset was performed applying a newly developed supervised machine learning software, the Advanced Cell Classifier (http://acc.ethz.ch). Based on this analysis, we obtained for each siRNA and readout a numerical score, termed hit rate, that was defined by the ratio of cells displaying phenotypes indicative of defects in ribosome synthesis to all reporter-positive interphase cells (Figure S2 and Text S1). SiRNAs causing a strong defect on cell growth were excluded from subsequent analysis.

**Figure 2:**
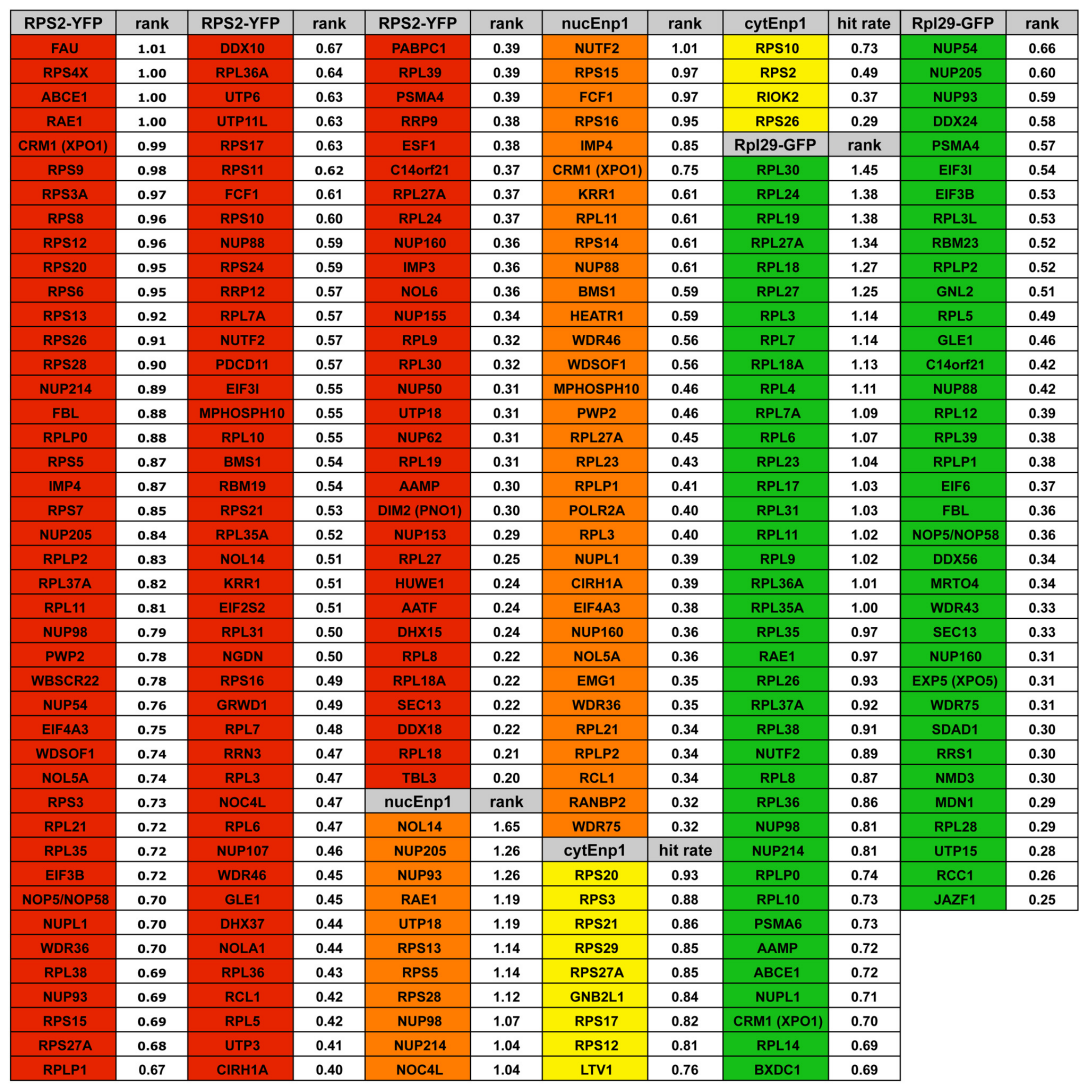
Ranked high confidence hit list. Compilation of high confidence hits for the three different readouts, Rps2-YFP, Enp1 IF, and Rpl29-GFP. Rank calculation was as follows: [(hit rate siRNA-X1)−(hit rate negative control)]/[(hit rate positive control)−(hit rate negative control)], where (hit rate negative control) and (hit rate positive control) equals the average hit rate of the two negative or positive controls with the highest hit rate from the same 96-well plate as siRNA-X1. For each hit, the average of the two highest ranked siRNAs is shown. For the Rps2-YFP readout, overall nuclear accumulation (nucleoplasmic and nucleolar) of the reporter was used for hit definition. Note that for “cytoplasmic” Enp1, absolute hit rates are given (ranking could not be performed due to a lack of an appropriate positive control for cytoplasmic accumulation). We set a cutoff to >0.2 for Rps2-YFP, >0.3 for Enp1 IF, and >0.25 for Rpl29-GFP (see Text S1).

Based on the hit rates, we further selected all targets, which were represented by at least two siRNAs resulting in more than twice the hit rate of the negative controls. Subsequently, these targets were ranked relative to both the negative and positive controls of the respective 96-well plate (for details, see Text S1). Next, we defined a cutoff (see Text S1), generating a high confidence hit list of targets that are involved in ribosome biogenesis based on the phenotypic classification of the different readouts (Figure 2). Lists of all numerical data are given in Tables S2, S3, and S4. All primary images are stored in a newly developed database, accessible via a web browser interface at http://hcpb.ethz.ch. We note that this database has all the required features to provide a framework for integrating future high content RNAi screening results.

### Proteins Required for Efficient Ribosome Synthesis in Human Cells

**Figure 3:**
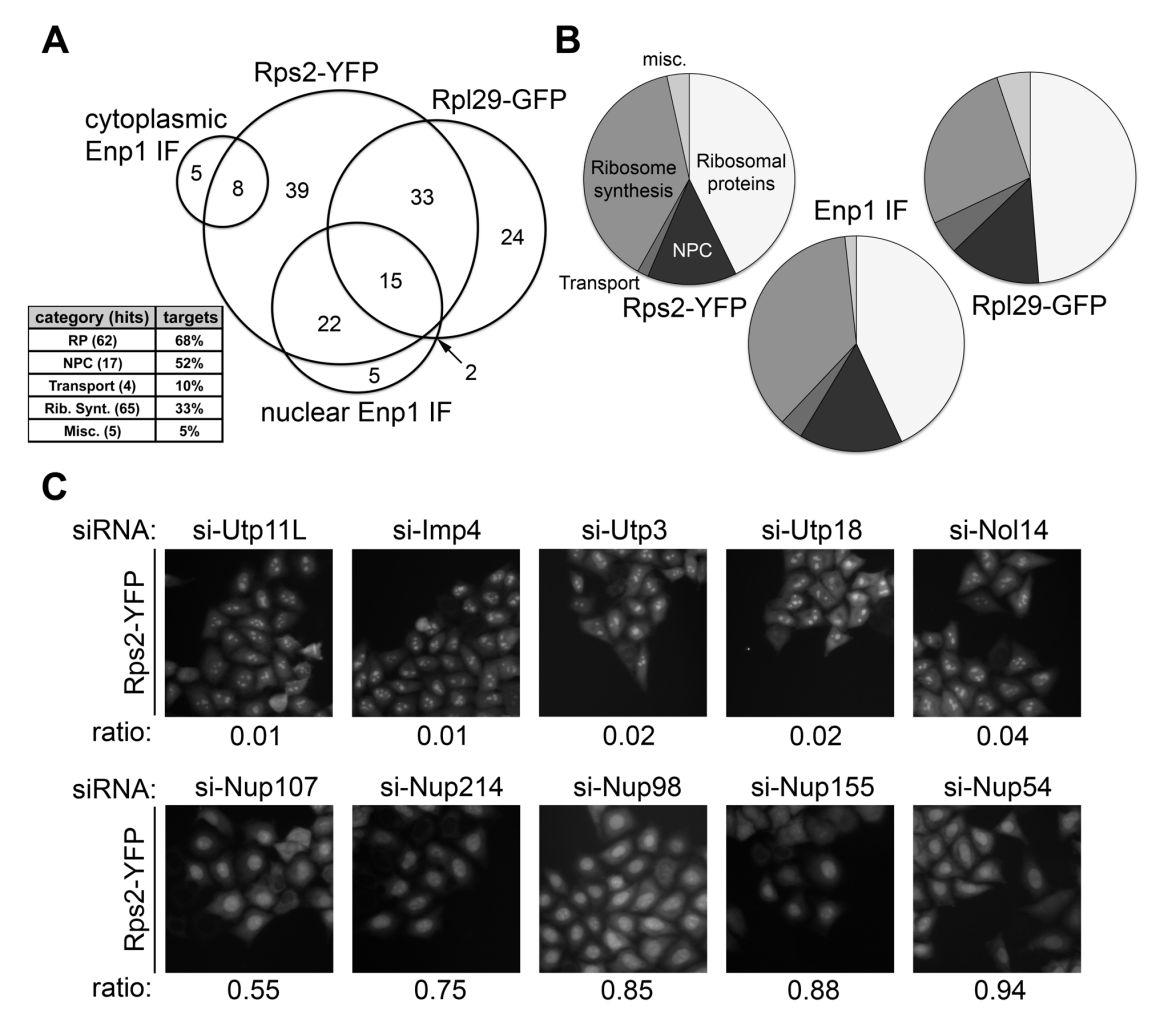
Hit distribution and example images of nucleolar and nucleoplasmic accumulation of Rps2-YFP. (A) Venn diagram of the 153 high confidence hits (Figure 2) and hit enrichment in the different target categories for all readouts (inset). (B) Distribution of high confidence hits to the different categories for each readout. (C) Phenotypic classification of targets with respect to different nuclear 40S biogenesis defects based on nucleolar or nucleoplasmic accumulation of Rps2-YFP. Classification was performed using the ratio of cells with a “nucleoplasmic” phenotype to all cells with a nuclear phenotype (nucleolar and nucleoplasmic). A value of 0 equals 100% of the hit-classified cells have a “nucleolar” phenotype, and a value of 1 equals 100% “nucleoplasmic” hits. Representative images after depletion of selected human SSU processome components (Utp11, Utp3, Imp4, Utp18, Nol14) and NPC components (Nup107, Nup98, Nup54, Nup214, Nup155) are shown.

Altogether, we identified 153 targets that displayed a phenotype in one or several of our readouts (Figure 2). A Venn diagram of these high confidence hits (Figure 3A) shows that there is a large coherence for the 40S readouts, with 84% of nuclear Enp1 IF hits also scoring as Rps2-YFP hits. Interestingly, there is also a significant fraction of targets common to nuclear steps in both 40S and 60S biogenesis (see below). Hits shared between all readouts are highly enriched for nucleoporins, the constituents of NPCs. Analysis of the hit distribution with respect to the functional categories of targets revealed that, as expected, ribosomal proteins were retrieved to a large extent, whereas only very few hits were found within the miscellaneous category, further illustrating the specificity of the used readouts (Figure 3A, B).

To test the reproducibility of the screening results, we performed two tests. First, the screen was repeated for a random subset of plates (∼50%). This analysis verified the high quality of the hit annotation, as 88% of the included high confidence hits for Rps2-YFP, 86% for Enp1, and 88% for Rpl29-GFP were confirmed (unpublished data). Notably, original hits that were not reproduced mostly turned up either just below the cutoff or were confirmed by only one siRNA during repetition. Second, to address whether the siRNA concentration was limiting, we repeated the whole analysis for the Rps2-YFP readout at an siRNA concentration of 25 nM (Figure S3). Importantly, this analysis confirmed 93% of our hits obtained at 10 nM. Although we obtained more hits at 25 nM, the higher siRNA concentration caused stronger effects on cell growth and likely increased off-target effects.

#### Nuclear 40S maturation

The Rps2-YFP readout allowed us to distinguish between nucleolar and nucleoplasmic defects in 40S subunit maturation. To delineate the contribution of individual factors along the nuclear 40S maturation pathway, we determined a numerical score for each target by calculating the ratio of cells displaying nucleoplasmic Rps2-YFP accumulation to all cells with a phenotype (nucleoplasmic and nucleolar) (Figure 3C). All targets were then sorted by their score on an ascending scale from 0 to 1 (Table S5). Low values (approaching 0) are indicative of nucleolar defects, whereas high values (approaching 1) reflect nucleoplasmic accumulation of the Rps2-YFP reporter. The power of this analysis is demonstrated by the fact that various factors that are human homologs of subunits of the yeast nucleolar SSU processome complex [10], which is involved in early subunit maturation, indeed have low scores. In contrast, NPC components required for subunit export are clustered at the high, “nucleoplasmic” end of the scale (Figure 3C). Overall, the majority of targets have low scores, with 82% of targets having predominantly “nucleolar” classified cells, i.e. scores below 0.5 (Table S5). Thus, most of the identified factors are required for 40S maturation in the nucleolus. Likewise, early stages of ribosome biogenesis in yeast are known to depend on the largest fraction of *trans*-acting factors [7].

#### 40S biogenesis responds to 60S biogenesis defects

The biggest group of hits consists of ribosomal proteins (RP). For the Rps2-YFP readout, ribosomal proteins of the small subunit (RPS) are numerous and the highest ranked; likewise, for the Rpl29-GFP readout, the highest ranked hits are ribosomal proteins of the large subunit (RPL). Interestingly, depletion of various RPL also impaired 40S biogenesis. In comparison, depletion of RPS only weakly affected 60S biogenesis, as indicated by the lack of RPS in the Rpl29-GFP hit list (Figure 2). In yeast, the early precursor for both subunits (90S particle) was shown to contain both RPS and 40S biogenesis factors, whereas RPL and 60S biogenesis factors were virtually absent [9]. Therefore, it seems counterintuitive that depletion of human RPS does not severely affect 60S biogenesis. However, this apparent weak sensitivity of 60S biogenesis to loss of individual RPS is consistent with a previous report showing that depletion of Rps6 in mouse blocks 40S biogenesis but leaves 60S biogenesis unaffected [47]. In *S. cerevisiae*, depletion of RPS has a very strong effect on 18S rRNA production, whereas 25S rRNA biogenesis is significantly less affected [48]. Also data on individual *trans*-acting factors in yeast support the conclusion that defects in 60S production have a stronger impact on 40S biogenesis than vice versa [49],[50], in agreement with our global analysis in human cells.

Taken together, these observations indicate that a subunit imbalance caused by 60S shortage is more deleterious to the cell than a 40S deficit. Possibly, subunit imbalance in the cytoplasm, i.e. decreased levels of 60S subunits, generates a signal relayed into the nucleus to tune down de novo synthesis of 40S. A plausible trigger may be the accumulation of mRNA-bound 40S subunits that are unable to complete translation initiation due to insufficient amounts of free 60S subunits. In the future, it will be interesting to learn which mechanism(s)—other than the processing from a common pre-rRNA transcript—might contribute to maintain an optimal subunit balance.

#### RPS in 40S assembly

Nomura and colleagues have defined the order of RPS assembly onto *E. coli* 16S rRNA in vitro [1] and classified RPS into primary binders, which bind to free 16S rRNA, secondary binders that depend on primary ones for 16S rRNA association, and tertiary binders that require both primary and secondary ones for their recruitment [51]. In yeast, RPS requirement for 40S biogenesis has been studied by monitoring specific rRNA processing defects in vivo [48]. Accordingly, yeast RPS were arranged into four groups, reflecting their involvement in different steps in nuclear biogenesis (I and II), in cytoplasmic maturation (III), and those without cytoplasmic processing block (IV). This RPS grouping revealed a striking accordance with the Nomura classification, thus establishing the conservation of RPS requirement during SSU assembly from *E. coli* to yeast.

**Figure 4:**
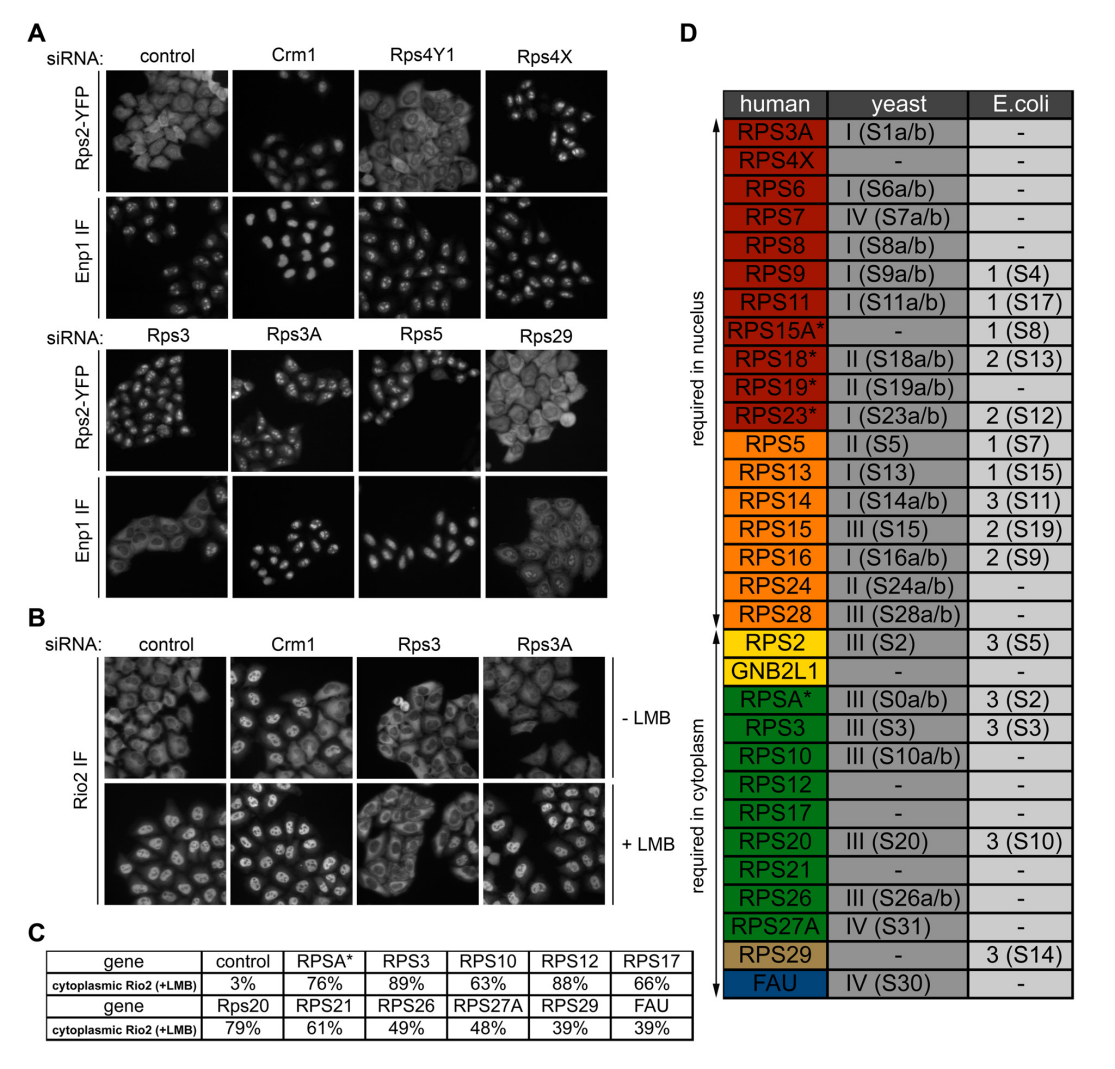
Requirement of small ribosomal proteins at different stages of 40S biogenesis. (A) Representative images from Rps2-YFP and Enp1 IF screening analysis after depletion of RPS by RNAi. Positive control: Crm1; negative controls: Allstars (Qiagen) and Rps4Y1 (Y-chromosome encoded). Rps3 depletion is shown as an example of differences detected with the Rps2-YFP (nuclear accumulation) and Enp1 IF (cytoplasmic accumulation) readouts. (B) Rio2 localization after depletion of RPS by RNAi (10 nM siRNAs, 72 h) in the absence or presence of 10 nM leptomycin B (LMB) for 2 h prior to fixation. Endogenous Rio2 was detected by immunofluorescence. Representative images are given for control, Crm1, Rps3, and Rps3A depletion. (C) List of RPS proteins that caused defects in nuclear accumulation of Rio2 upon LMB treatment when downregulated by RNAi in (B). An RPS was defined as a hit if more than 20% of cells were classified as “cytoplasmic” Rio2 after LMB treatment. The average of the two highest scoring siRNAs is given. *For RspA, only 1 siRNA gave a phenotype. (D) RPS were sorted according to the phenotypes detected in the Rps2-YFP, Enp1 IF, and Rio2 IF readouts and grouped with respect to their nuclear or cytoplasmic requirement for human 40S biogenesis. “Nuclear” classified RPS were either detected with Rps2-YFP only (red) or with both the Rps2-YFP and Enp1 IF readouts (orange). Classification of “cytoplasmic” RPS is based on Enp1 IF (yellow), Rio2 IF (+LMB) (brown), or both (green; depletion of these RPS also caused nuclear Rps2-YFP accumulation). FAU was scored as a nuclear Rps2-YFP hit and a cytoplasmic Rio2 IF (+LMB) hit (blue). RPS marked by an asterisk scored positive for one siRNA by at least 7 times the hit rate of negative controls. The yeast RPS classification into four groups (I–IV) is based on [48] and described in the text. For *E. coli* RPS, primary (1), secondary (2), and tertiary (3) binders of the Nomura assembly map [1],[51] are listed. The names of yeast and bacterial homologs are given in brackets. FAU and GNBL2L1 are the human ribosomal proteins Rps30 and RACK1, respectively.

The datasets relying on the Rps2-YFP and Enp1 reporters allowed us to define at which stage the absence of a specific RPS interfered with 40S biogenesis in human cells. In this analysis, 18 RPS clearly caused a nuclear 40S biogenesis defect (Figure 4A, D).

For the remaining RPS, a few were classified as hits that caused nuclear Rps2-YFP accumulation but cytoplasmic enrichment of Enp1. Because cytoplasmic Enp1 localization in principle could also stem from Enp1 not bound to pre-40S subunits, we additionally monitored the localization of another *trans*-acting factor, Rio2, that allows for a second readout on cytoplasmic subunit maturation. Rio2, which is a stable component of late, cytoplasmic pre-40S [45], appears cytoplasmic at steady state, but rapidly accumulates in the nucleus upon inhibition of the export receptor Crm1 by the drug leptomycin B (LMB) ([45] and Figure 4B). However, when pre-40S subunits are stalled in their cytoplasmic maturation, Rio2 is trapped on these particles in the cytoplasm and fails to relocalize to the nucleus upon inhibition of Crm1 (Figure 4B, Rps3 RNAi ± LMB). Using this approach, we confirmed that all RPS, which had caused a cytoplasmic Enp1 phenotype upon depletion, were indeed required for cytoplasmic pre-40S maturation (Figure 4B, C). The nuclear export defect observed using the Rps2-YFP readout upon depletion of these particular RPS might be caused by a kinetic delay in export because the Rps2-YFP readout readily senses kinetic delays in nuclear subunit maturation [45].

When we compile these data and classify human RPS according to their requirement for nuclear or cytoplasmic 40S maturation (Figure 4D), we find a striking agreement with the yeast data, except for Rps7, which we classified as a “nuclear” hit but was assigned to group IV in yeast. All human homologs of yeast groups I and II RPS score as “nuclear” hits, whereas those corresponding to yeast group III RPS are predominantly found among the “cytoplasmic” hits. The respective human RPS of group IV also score “cytoplasmic”. When compared to bacterial ribosome formation, human RPS that correspond to *E. coli* primary and secondary binders cluster as nuclear hits, whereas homologs of tertiary binders are “cytoplasmic”. We confirmed our classification for selected RPS by Northern blot analysis of rRNA processing (Figure S4). Moreover, our data fit well with recent data on the effects of depletion of a subset of human ribosomal proteins on rRNA processing [52]. Thus, the specific requirement for individual ribosomal proteins of the SSU along the 40S maturation pathway is conserved from *E. coli* to man.

#### *Trans*-acting factors involved in subunit synthesis

For all our readouts, many targets that were identified as hits belong to the ribosome biogenesis category based on their homology to yeast *trans*-acting factors. Hence, our dataset establishes a functional conservation of these human proteins in ribosome synthesis. Altogether, 65 factors of this class caused severe defects in ribosome synthesis when downregulated (Figure 3A). For example, the Rps2-YFP and Enp1 hit lists (Figure 2) are enriched for human homologs of yeast 40S *trans*-acting factors, such as BMS1, PNO1, and WBSCR22, corresponding to yeast *trans*-acting factors Bms1, Dim2, and Bud23, respectively. Likewise, the Rpl29-GFP hit list contains human homologs of yeast 60S *trans*-acting factors, such as Mrt4 (MRTO4), Sqt1 (AAMP), or Rea1 (MDN1). Thus, the pathway-specific role of these factors is conserved in humans. However, we note that depletion of 60S biogenesis factors can also affect the 40S biogenesis pathway as observed for Sqt1 (AAMP)—in agreement with the finding that knockdown of RPL proteins influences 40S formation. Generally, a functional conservation of factors between yeast and human as revealed by our dataset may also motivate efforts to understand their molecular role in more detail.

#### Nucleo-cytoplasmic transport

Like in yeast [18]–[20], a large group of hits are nucleoporins. Many of the nucleoporin hits are FG repeat-containing nups directly involved in nuclear transport such as Nup62, Nup54, Nup50, Nup98, Nup214, and Nup153. The dependence of ribosomal biogenesis on both the nuclear export of subunits and the import of ribosomal proteins and *trans*-acting factors also implies an important role for the RanGTPase system in governing the directionality of these transport processes. This is supported by the occurrence of the Ran import receptor NTF2 (NUTF2) and the RanGEF RCC1 in our hit list. Unexpectedly, besides Crm1, we also identified a second RanGTP-binding exportin, exportin 5, among the factors affecting 60S biogenesis (see below).

#### Hits in the miscellaneous category

Among the 103 targets compiled in the miscellaneous category, only 5 were retrieved as hits in our screen. Notably, two of them, eIF3B and eIF3I, are subunits of the eIF3 translation initiation complex and another two, PMSA4 and PMSA6, are constituents of the 20S proteasome. Follow-up analysis revealed that several other subunits of both eIF3 and the proteasome, which were not included in our screen, are also required for human ribosomal subunit maturation (unpublished data). Hence, the function of the proteasome and the eIF3 complex seem important for human ribosome biogenesis. These observations not only extend previous reports on connections between ribosome synthesis and protein degradation [53],[54], but also are in line with a recently described link between the eIF3 complex and ribosome biogenesis in *S. pombe* [55].

### Exportin 5 Is Required for 60S Export

**Figure 5:**
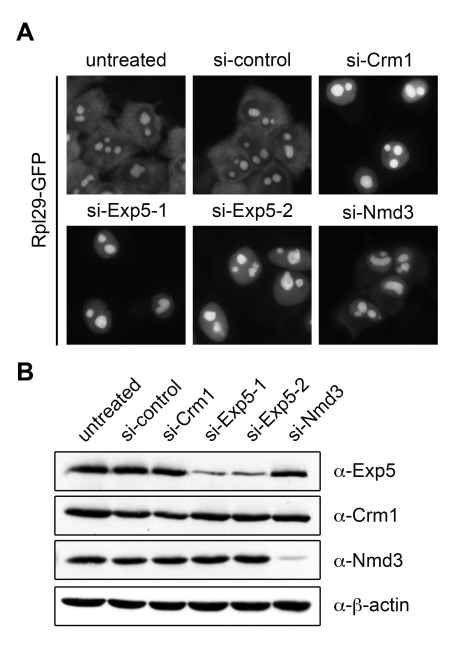
Depletion of Exportin 5 leads to a 60S export defect in human cells. (A) The Rpl29-GFP cell line was treated with the indicated siRNAs (15 nM) for 72 h. After 52 h of RNAi, Rpl29-GFP expression was induced for 14 h by addition of tetracycline, followed by incubation in tetracycline-free medium for 6 h and subsequent fixation. Images were taken by epifluorescence microscopy. (B) Western blot analysis of extracts from cells derived from the experiment shown in (A). Note that even a slight downregulation of Crm1 results in a prominent and highly reproducible 60S export defect, as also previously described for 40S export [45].

Exportin 5 (Exp5) was detected in the screen as a 60S-specific hit. Because only one siRNA gave rise to a robust phenotype in the screen, we verified the result using two previously validated siRNAs against Exp5 ([56], unpublished data). Depletion of Exp5 indeed resulted in nuclear accumulation of Rpl29-GFP (Figure 5), comparable to depletion of the positive controls Crm1 and Nmd3, which serves as an adaptor protein for Crm1 in 60S export [34],[35],[57],[58]. Northern blot analysis using an ITS2-specific probe that detects precursors of 28S rRNA did not reveal any rRNA processing defects induced by depletion of Exp5 (Figure S5).

Exp5 is an RNA-binding exportin that functions in nuclear export of miRNA precursors [56],[59],[60]. Recently, miRNA-10a was shown to positively regulate the expression of certain mRNAs, including mRNAs coding for ribosomal proteins of both the small and the large subunit [61]. These data showed that both 40S and 60S biogenesis were slightly compromised upon miRNA-10a inhibition. We therefore tested whether components of the miRNA biogenesis pathway would be required for 40S and 60S synthesis. In these experiments we found no evidence for a phenotype in nuclear 60S biogenesis similar to that caused by Exp5 depletion (Figures S6, S7). Depletion of Exp5 also gave rise to detectable defects in 40S biogenesis, as based on nuclear accumulation of the Rps2-YFP reporter (Figure S6, Table S2, and unpublished data). However, defects in 60S biogenesis were more prominent. Thus, there appears to be a stronger dependence of 60S than 40S biogenesis on the presence of Exp5. Taken together, an impairment of miRNA synthesis or function seemed insufficient to explain the strong 60S-specific phenotype for Exp5 depletion in our assay.

### Exp5 Specifically Binds 60S Particles in a RanGTP-Dependent Manner

**Figure 6:**
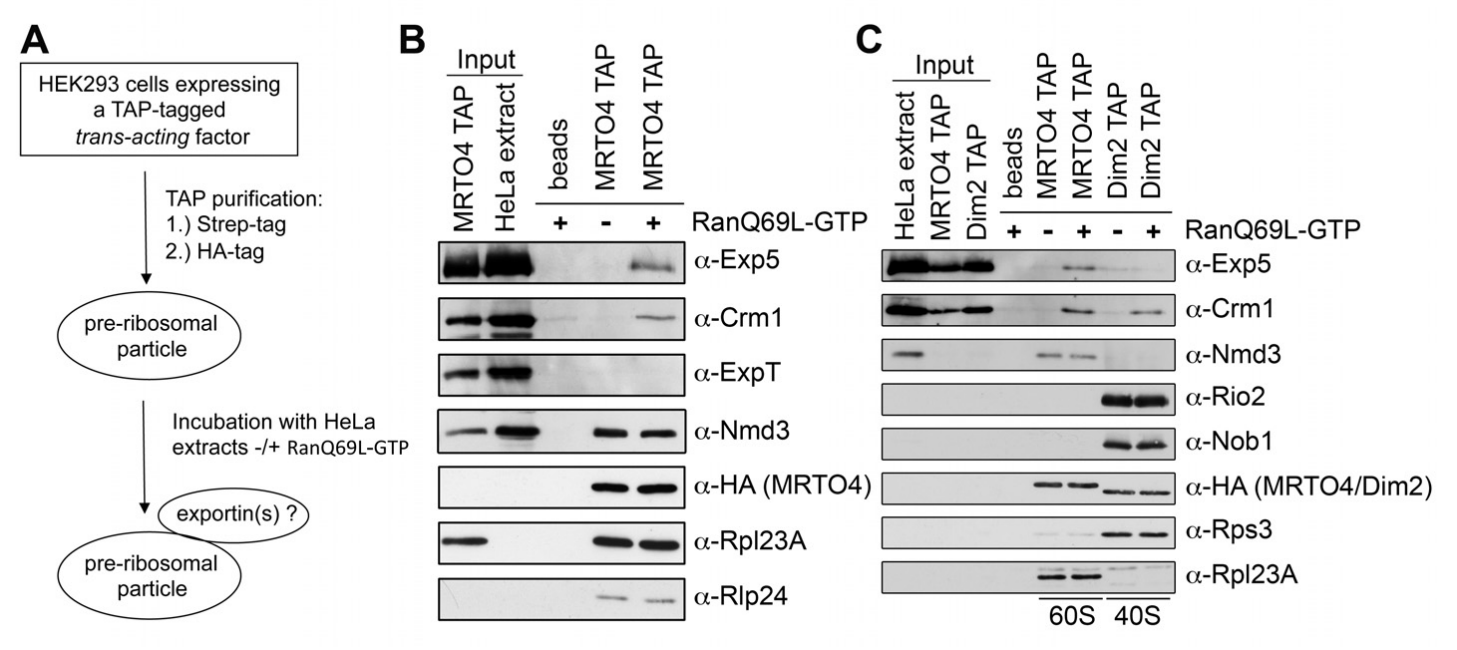
Exp5 binds specifically to pre-60S particles in a RanGTP-dependent manner. (A) Schematic representation of the experimental setup. Expression of a tagged bait protein in HEK293 cells was induced by tetracycline. Cells were lysed and the bait protein was affinity-purified by help of its tandem strep/HA-tag using a two-step protocol. Next, the bait protein and associated proteins, bound to anti-HA IgG beads, were incubated with HeLa extracts with or without addition of RanQ69L-GTP. Subsequently, bound proteins were analyzed by Western blotting. (B) Exp5 binds to pre-60S particles in the presence of RanGTP. Pre-60S particles were purified from HEK293 cells bearing an inducible copy of TAP-tagged MRTO4 after 24 h of tetracycline treatment (cell lysate: input MRTO4 TAP). The purified particles were incubated with HeLa cell extract (input HeLa extract) in the absence or presence of 10 µM RanQ69L-GTP. Anti-HA IgG beads were used as a control for non-specific binding of exportins in presence of RanQ69L-GTP. (C) Binding of Exp5 is specific for pre-60S particles. Experiment as in (B) including affinity purification of pre-40S subunits by Dim2 (PNO1) TAP from HEK293 cells bearing an inducible copy of TAP-tagged Dim2. Note that ribosomal particle components are not detected in the dilute input of HEK293 lysates but are highly enriched during purification. Load of input and bound fractions as in (B).

Therefore, we next addressed whether Exp5 could have a direct function in 60S export. An exportin/cargo relationship between Exp5 and pre-60S particles would predict a physical, RanGTP-regulated interaction between Exp5 and pre-60S particles [62]. To test this, we first purified a pre-60S particle from HEK293 cells by tandem affinity purification (TAP) using the TAP-tagged *trans*-acting factor MRTO4 (homologous to yeast Mrt4) as bait (Figure 6 and Figure S8). Protein composition analysis of this particle showed the presence of 60S *trans*-acting factors like the export adaptor Nmd3, C15orf15 (Rlp24, ribosomal-like protein 24), and ribosomal proteins of the large subunit (Figure 6B and Figure S8A). These particles were then incubated with HeLa cell extract in the absence or presence of RanQ69L-GTP, a Ran mutant locked in the GTP-bound state, to analyze RanGTP-dependent binding of nuclear export receptors to pre-60S. Note that the HeLa cell extract had been pre-depleted for ribosomes and tRNAs. The depletion of ribosomes ensured that the TAP-purified particles were the sole ribosomal particles present. The presence of tRNA might hamper the detection of a potential Exp5/60S interaction because tRNA binds efficiently to Exp5 [63],[64].

Reflecting their requirement for pre-60S export, both Exp5 and Crm1 bound to this pre-60S particle in a RanGTP-dependent manner, whereas exportin-t (Exp-t), another exportin that was not detected as a hit in the screen, did not bind (Figure 6B). We confirmed these results using a pre-60S particle purified with ZPR9 (REI1), another TAP-tagged 60S *trans*-acting factor (Figure S8B). The addition of competitor pre-miRNA to the binding reaction inhibited Exp5 binding but left Crm1 binding unaffected (Figure S8C), showing that Crm1 binds independently of Exp5 and indicating that Exp5 uses its RNA-binding interface for pre-60S particle association. Overall, the observed RanGTP-dependent binding of Crm1 and Exp5 to pre-60S TAP particles reflects a bona fide exportin/cargo interaction.

Since the depletion of Exp5 had also affected 40S biogenesis, albeit to a lesser extent than 60S synthesis, we analyzed whether Exp5 binding is 60S-specific or can also be observed for pre-40S particles. Pre-40S particles were similarly purified by TAP using the 40S *trans*-acting factor PNO1 (Dim2) as bait (Figure 6C). These pre-40S particles contain other expected 40S *trans*-acting factors such as Rio2 and Nob1, as well as 40S ribosomal proteins (Figure 6C and Figure S8A). Crm1 bound to this particle in a RanGTP-dependent manner and the particle can therefore be considered an export-competent pre-40S. In contrast, Exp5 did not bind to this pre-40S particle (or another purified pre-40S particle, unpublished data), further illustrating the specificity of 60S binding. These data substantiate a direct role for Exp5 in 60S export.

It should be pointed out that RanGTP-dependent binding of Crm1 to pre-40S and pre-60S particles has not been demonstrated previously. Thus, this analysis of exportin binding to pre-ribosomal subunits also provides the first biochemical evidence for the prevailing model of a direct role of Crm1 in the export of both ribosomal subunits.

### Exp5 Exports 60S Subunits in Vertebrates

**Figure 7:**
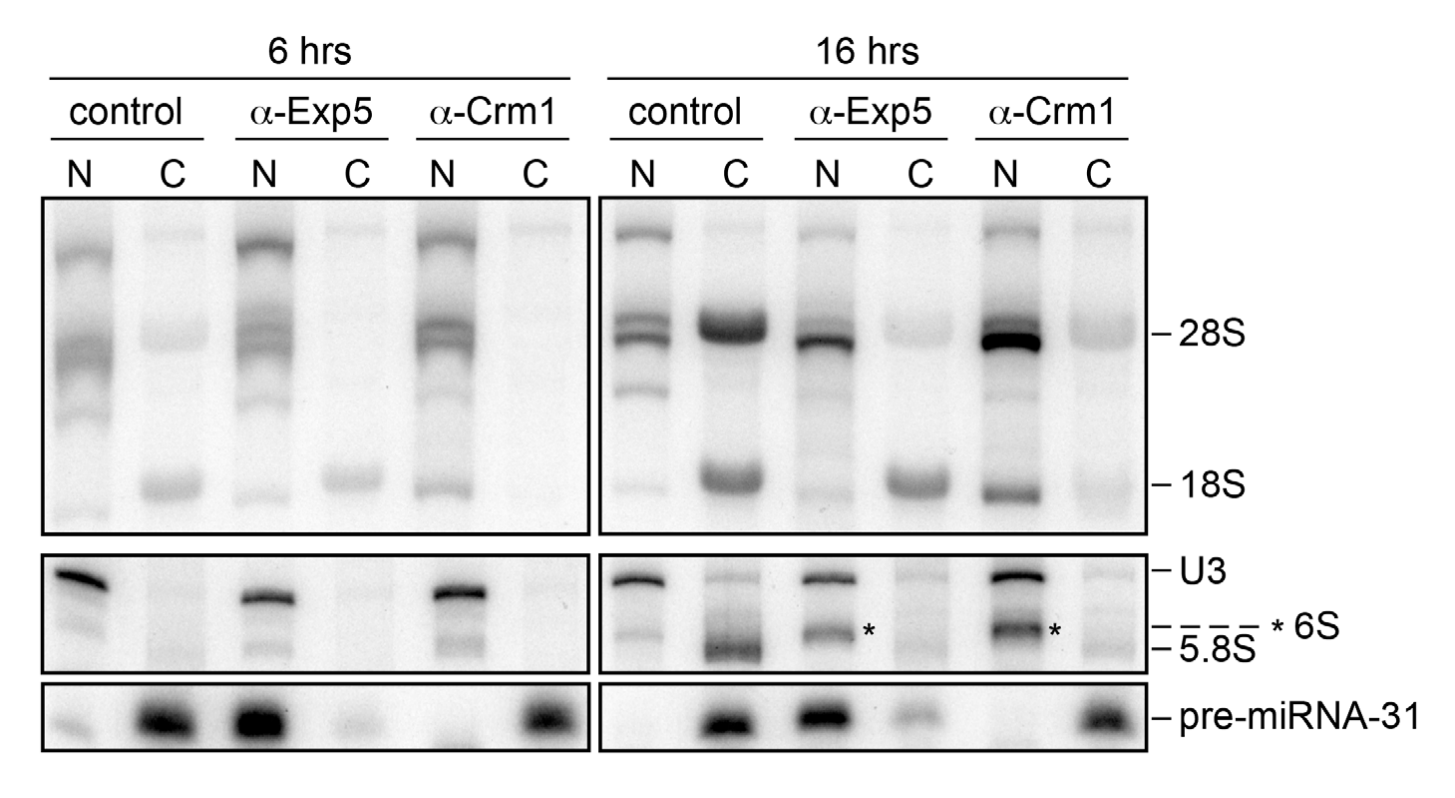
Exp5 is required for nuclear export of 60S subunits in frog oocytes. *X. laevis* oocytes were pre-injected in the nucleus with antibodies against Exp5 or Crm1 together with ^32^P-labeled U3 snRNA and pre-miR-31. To label newly synthesized rRNA, ∼0.5 µCi α-^32^P-GTP was injected into the cytoplasm 3 h later. After incubation for the indicated times, pools of oocytes were dissected into nuclear (N) and cytoplasmic (C) fractions, and total RNAs extracted. For analyses of large (top panel) or small RNAs (bottom panels), total RNAs were separated by electrophoresis in denaturing 1.2% agarose gels or 8% 7M Urea polyacrylamide gels, respectively. Dried gels were exposed to x-ray film for 20 h (top panel) or 3–4 d (bottom panels). Note the increased accumulation of 28S and 6S(pre-5.8S)(*) [35] rRNAs in the nucleus upon inhibition of pre-60S subunit export. U3 snRNA and pre-miR-31 serve as markers for nuclear injection/proper dissection and Exp5 activity, respectively.

To confirm the function of Exp5 in 60S export in another vertebrate species and by a different experimental approach, we analyzed the exportin dependence of rRNA export in *Xenopus* oocytes. Neutralizing antibodies specific for Crm1 or Exp5 were injected into oocyte nuclei, and subsequently the maturation and export of newly made, radio-labeled rRNAs were assessed after dissection of the oocytes into nuclear (N) and cytoplasmic (C) fractions (Figure 7). Under control conditions, various pre-rRNA species are detected in the nuclear fraction, whereas mature rRNAs of the 60S subunit (28S rRNA, 5.8S rRNA) and the 40S subunit (18S rRNA) are found in the cytoplasmic fraction. Injection of Crm1 antibodies resulted in nuclear accumulation of both 28S and 18S rRNAs, consistent with previous experiments [34],[35]. Upon injection of the Exp5 antibody, 28S rRNA and 6S rRNA, the precursor to 5.8S rRNA, accumulated in the nucleus, but 18S rRNA export was unaffected. Thus, Exp5 is the second exportin besides Crm1 required for 60S export in higher eukaryotes.

In yeast, deletion of Msn5, the homolog of Exp5, does not impair ribosomal subunit export [18],[19], indicating that pre-60S export is different between yeast and man. Moreover, recent studies have identified two additional export factors besides Crm1 that contribute to 60S export in yeast: the general mRNA export receptor Mex67/Mtr2 and the *trans*-acting factor Arx1 [65]–[67]. The domains in Mex67/Mtr2 and Arx1 required for pre-60S binding and NPC interaction, respectively, are not conserved from yeast to humans; hence, their human homologs are not expected to contribute to 60S export [65],[67]. Notably, we did not detect the human homolog of Mex67 (Tap, NXF1) on our purified pre-60S particles (Figure S8B) and also found no evidence for the involvement of Tap or EBP1 (Arx1) in 60S biogenesis in our screen. However, we note that in yeast the depletion of Arx1 alone causes no 60S export defect [65].

Thus, at least two export receptors are required for 60S export in both humans and yeast. Here, we demonstrate for the first time the binding of two exportins to a pre-60S particle. Importantly, neither receptor on its own is sufficient to promote 60S export in vivo (Figure 7). Together, these data suggest that a particle as big as a 60S pre-ribosome requires more than one transport receptor for NPC passage. The usage of the pre-miRNA export receptor Exp5 for 60S subunit export opens the possibility for crosstalk between miRNA biogenesis and 60S export, but it remains to be seen whether cells make use of this. In yeast, a crosstalk between mRNA and 60S export has been proposed based on the shared usage of the export receptor Mex67/Mtr2 [68].

### Conclusion and Perspective

We present a first, partial protein inventory for human ribosome biogenesis. The scope of this RNAi screen was to evaluate candidate genes for human ribosome synthesis, building largely on the knowledge about eukaryotic ribosome biogenesis obtained in yeast. We provide a list of 153 proteins (including 91 non-ribosomal proteins) that are required for ribosome synthesis in human cells. Moreover, both primary and analyzed data of all investigated targets can be accessed through an online database (http://hcpb.ethz.ch). Additionally, we demonstrate a direct requirement for the RanGTP-binding transport receptor Exp5 in the biogenesis of 60S subunits in vertebrates.

The presented RNAi screen can be extended in future to perform unbiased genome-wide searches for additional factors that participate in ribosome biogenesis. We anticipate that many features of the process in vertebrates will be found to be analogous to those in yeast, but differences have already been identified. These include proteins that participate in ribosome biosynthesis in yeast or humans and are not conserved between yeast and vertebrates, for example vertebrate nucleophosmin [69]–[71], as well as the many uncharacterized proteins present in human nucleoli [72],[73]. Moreover, regulation of ribosome synthesis is likely to be more complex in vertebrates than in unicellular organisms.

Systematic RNAi together with complementing approaches might ultimately lead to a comprehensive inventory of proteins involved in this fundamental process in mammalian cells and may pave the way towards a deeper mechanistic knowledge. Given the emerging evidence for numerous links between human diseases and ribosome biogenesis/function [74]–[76], such progress is eagerly awaited.

## Material and Methods

### Cell Lines

HeLa Rps2-YFP has been described [45]. The HeLa Rpl29-GFP cell line was generated by integrating Rpl29-GFP (cloned into the KpnI and NotI sites of pcDNA/FRT/TO; Invitrogen) into HeLaK FRT TetR cells [45].

### Screening

siRNAs (10 µl of a 100 nM stock in OptiMEM; Invitrogen) were added to the transfection reagent (0.0625 µl Oligofectamin (Invitrogen) in 20 µl OptiMEM) in wells of 96-well plates and incubated at RT for 30 min. Then, 70 µl of cells were added to each well (1,750 cells for HeLa Rps2-YFP and HeLa Rpl29-GFP cell lines, 1,250 cells of HeLa cells for Enp1 IF). 58 h after transfection, Rps2-YFP cells were induced with tetracycline (final concentration of 125 ng/ml) for 14 h. Rpl29-GFP cells were treated similarly, except induction with tetracycline was followed by incubation in tetracycline-free medium for 6 h. HeLa cells (used for Enp1 IF analysis) were incubated for 72 h after siRNA transfection. Cells were fixed with 4% PFA and DNA stained with Hoechst. Enp1 IF was performed as described [45]. Cells were automatically imaged using a 20× objective on a BD pathway 855 microscope.

The Allstars Negative Control (Qiagen) was included three times on each plate. All siRNAs used for screening were designed by and purchased from Qiagen.

### Access to Screening Data

All images and numerical data are accessible at http://hcpb.ethz.ch.

### TAP Purification and Exportin Binding

Generation of HEK293 cell lines expressing TAP-tagged proteins and subsequent purification has been described [77]. HeLa extracts depleted of ribosomes by ultracentrifugation [78] and of tRNA [79] were added to purified particles without or with the addition of 10 µM RanQ69L-GTP. Binding reactions were performed in 50 mM Tris/HCl pH 7.5, 150 mM KOAc, 2 mM MgCl_2_, and 0.001% Triton X-100.

### Antibodies

Antibodies against the following human proteins have been described: Enp1, Nmd3, Nob1, Rio2 and Rps3 [45], Crm1, hExp5 [56], and Exp-t [80]. The α-Rlp24 antibody was generated in rabbits using 6His-tagged Rlp24 as antigen. Anti-β-Actin (Sigma-Aldrich) and α-HA IgG beads (Sigma) are commercially available. Anti-*Xenopus* Exp5 antibodies were a kind gift from D. Görlich [59].

### siRNAs

A custom assembled siRNA library was purchased from Qiagen. For individual analysis, RNAi was performed as described above, but in 6 wells. SiRNAs (sense): Crm1 (5′-UGUGGUGAAUUGCUUAUAC), Exp5-1 (5′-AGAUGCUCUGUCUCGAAUU), Exp5-2 (5′-UGUGAGGAGGCAUGCUUGU) [59], and Nmd3 (5′-GAAUGGUGCUAUCCUUCAA).

### *Xenopus* Oocyte Microinjection

^32^P-labeled RNAs encoding U3 snoRNA and pre-miR-31 were transcribed in vitro from PCR templates using α-[^32^P]-GTP as previously described [81]. The microinjection and dissection of *Xenopus* oocytes and ^32^P-labeling and analysis of rRNAs were performed according to [35].

## Supporting Information

Figure S1 **Rpl29-GFP is incorporated into 60S subunits.** Extract from HeLa Rpl29-GFP expressing cells (induced with 125 ng/ml tetracycline for 14 h followed by 6 h chase in tetracycline-free medium) was separated on a 10% to 45% sucrose gradient (50 mM Hepes/KOH pH 7.5, 100 mM KCH_3_CO_2_, 3 mM MgCl_2_). Protein in gradient fractions was precipitated and analyzed by immunoblotting using the indicated antibodies.
(0.39 MB TIF)

Figure S2 **Confusion matrices of phenotypic classification.** (A) Cross-validation of visual and computational cell classification for HeLa Rps2-YFP cells. Cells were visually classified into the following predefined phenotypes: cytoplasm (prominent cytoplasmic Rps2-YFP signal), nucleoplasm (predominant nucleoplasmic Rps2-YFP signal), nucleolus (predominant nucleolar Rps2-YFP signal), no Rps2-YFP (cells not expressing Rps2-YFP), mitotic (cells in mitosis), and debris (cell debris; image segmentation errors). Subsequently, computational classification was performed based on the visual classification. The confusion matrix shows the degree of accordance between visual and computational classification. Absolute numbers of classified cells are given in brackets. As a measure for the quality of computational classification, the ROC (receiver operating characteristic) area was calculated. Note that an ROC area >0.9 is considered as excellent. (B) Cross-validation of visual and computational cell classification for cells analyzed by Enp1 IF cells. Predefined phenotypes are: nucleolus (predominantly nucleolar Enp1 IF signal), nucleoplasm (homogenous nuclear Enp1 IF signal), cytoplasm (prominent cytoplasmic Enp1 IF signal), mitotic (cells in mitosis), and debris (cell debris or image segmentation errors). Note that no debris was present in the training set for this readout. (C) Cross-validation of visual and computational cell classification for HeLa Rpl29-GFP cells. Predefined phenotypes are: cytoplasm (nucleolar and cytoplasmic Rpl29-GFP signal), nucleus (nucleolar and nucleoplasmic Rpl29-GFP signal), no Rpl29-GFP (cells not expressing Rpl29-GFP), mitotic (cells in mitosis), and debris (cell debris or image segmentation errors).
(0.57 MB TIF)

Figure S3 **List of additional targets identified as hits at 25 nM siRNA concentration using the Rps2-YFP readout.** High confidence hits from Rps2-YFP screen at 25 nM, which were not detected in the screen at 10 nM. Ranking was performed as described.
(0.21 MB TIF)

Figure S4 **Depletion of different RPS causes distinct rRNA processing defects.** RNA isolated from HeLa cells treated with the indicated siRNAs (15 nM) for 72 h was analyzed by Northern blotting using a 5′-ITS1 specific probe [31]. Note that RpsA depletion caused accumulation of a nuclear rRNA species (30S). 18S-E rRNA, which is both nuclear and cytoplasmic, accumulated upon depletion of Rps15 and Rps3. The accumulation of 18S-E rRNA is more pronounced upon depletion of Rps3, a RPS classified to be required for cytoplasmic 40S maturation.
(0.78 MB TIF)

Figure S5 **Depletion of Exp5 does not cause rRNA processing defects in the 28S rRNA processing pathway detectable by the ITS2 probe.** (A) HeLa Rpl29-GFP cells were treated with the indicated siRNAs (15 nM). After 52 h of RNAi, Rpl29-GFP expression was induced for 14 h by addition of tetracycline, followed by incubation in tetracycline-free medium for 6 h and subsequent RNA extraction. Isolated RNA was analyzed by Northern blotting using an ITS2-d/e [31] specific probe. Ethidium bromide staining of 18S and 28S rRNA is shown as loading control. Note that Exp5 depletion caused no visible rRNA processing defects (similar to Crm1 RNAi), whereas rRNA processing defects are observed upon Rpl3 RNAi (aberrant rRNA species marked with *). Allstars siRNA (Qiagen) was used as a negative control. (B) As a phenotypic readout for efficient RNAi, images of HeLa Rpl29-GFP cells from experiment shown in (A) were taken by epifluorescence microscopy.
(0.65 MB TIF)

Figure S6 **Analysis of 40S and 60S biogenesis defects upon RNAi against miRNA biogenesis factors.** (A) HeLa Rps2-YFP cells were treated with the indicated siRNAs (10 nM). RNAi against Crm1 served as positive control, and the Allstars siRNA (Qiagen) was used as a negative control. After 58 h of RNAi, Rps2-YFP expression was induced for 14 h by addition of tetracycline and cells were subsequently fixed. Images were taken by the screening microscope (BD pathway 855). (B) HeLa Rpl29-GFP cells were treated with the indicated siRNAs (10 nM). After 52 h of RNAi, Rpl29-GFP expression was induced for 14 h by addition of tetracycline, followed by incubation in tetracycline-free medium for 6 h and subsequent fixation. Images were taken by the screening microscope (BD pathway 855). Note that for Exp5, only siRNA 2 was also used in the original screening analysis. SiRNA 1 and siRNA 3 are additional, independent siRNAs not used in the original screening and also different from those used in Figure 5.
(6.21 MB TIF)

Figure S7 **Analysis 60S biogenesis defects after depletion of miRNA biogenesis factors.** (A) HeLa Rpl29-GFP cells were treated with the indicated siRNAs (10 nM). After 52 h of RNAi, Rpl29-GFP expression was induced for 14 h by addition of tetracycline, followed by incubation in tetracycline-free medium for 6 h and subsequent fixation. Images were taken by epifluorescence microscopy. (B) Western blot analysis of extracts from cells derived from the experiment shown in (A). Enp1 levels were used as loading control. (C) Experiment as described in (A) with indicated siRNAs. (D) Western blot analysis of extracts from cells derived from the experiment shown in (C). Enp1 levels and a weak unspecific band served as loading controls. (E) Experiment as described in (A) with indicated siRNAs. (F) Western blot analysis of extracts from cells derived from the experiment shown in (A). Enp1 levels were used as loading control.
(3.62 MB TIF)

Figure S8 **Exportin 5 binding to pre-60S particles isolated by tandem affinity purification.** (A) Silver-stained PAA-SDS gel of pre-60S and pre-40S particles isolated on TAP-tagged MRTO4 and Dim2, respectively. Large ribosomal proteins (RPL) and small ribosomal proteins (RPS) isolated on TAP-tagged MRTO4 and Dim2, respectively, are indicated. (B). Exportin 5 binds to pre-60S particles isolated on two different TAP-tagged pre-60S-associated *trans*-acting factors. Pre-60S particles were purified from HEK293 cells (Input ZPR9-TAP, MTRO4-TAP) bearing either an inducible copy of TAP-tagged MRTO4 or TAP-tagged ZPR9 24 h after tetracycline addition. The purified particles were incubated with tRNA-depleted, postribosomal HeLa cell extract (input HeLa extract) in the absence or presence of 10 µM RanQ69L-GTP. RanQ69LΔC, which cannot be dissociated from its cargo, was used in the sample marked with an asterisk and enhances both Exp5 and Crm1 retrieval. Anti-HA IgG beads were used as a control for unspecific binding of exportins in the presence of RanQ69L-GTP. (C) Pre-miRNA competes for RanGTP-dependent binding of Exportin 5 to pre-60S particles. Experiment was performed essentially as in (B). The pre-miRNA competition experiment was performed by addition of in vitro transcribed pre-miRNA-31 [56] to a final concentration of 10 µM.
(0.25 MB TIF)

Table S1 **List of targets in the systematic analysis of ribosome biogenesis by RNAi.** Targets are sorted according to the following categories: ribosomal proteins, NPC components, nuclear transport, ribosome synthesis, and a miscellaneous group. For proteins of the “ribosome synthesis” category, if applicable, a reference linking the homologous yeast protein to ribosome biogenesis is given.
(0.10 MB XLS)

Table S2 **Summary of numerical data derived from the Rps2-YFP screen.** The screen was performed in 26 96-well plates. Each 96-well plate contained three negative controls, three positive controls (Xpo1/Crm1 siRNA), and three siRNAs per target gene. For each siRNA (well), the following information is given: plate number (96-well plate identity), cell number (based on Hoechst staining), relative cell number (cell number in the respective siRNA containing well divided by average cell number of all three negative controls of same 96-well plate), YFP-positive cells (interphase cells expressing Rps2-YFP), hit-classified cells (cells classified as “nucleolar” or “nucleoplasmic” Rps2-YFP phenotype), hit rate (ratio of hit-classified cells to YFP-positive cells), and nucleoplasmic/nucleolar (ratio of “nucleoplasmic” to hit-classified cells). Note that for Rpl23A only two siRNAs were used.
(0.13 MB XLS)

Table S3 **Summary of numerical data derived from the Enp1 IF screen.** For each siRNA, the following information is given: plate number (96-well plate identity), cell number (based on Hoechst staining), relative cell number (cell number in the respective siRNA containing well divided by average cell number of all three negative controls of same 96-well plate), interphase cells, “nucleoplasmic” (cells displaying enhanced nucleoplasmic localization of Enp1), hit rate (nuc.) (ratio of “nucleoplasmic” cells to interphase cells), “cytoplasmic” (cells displaying cytoplasmic localization of Enp1), and hit rate (cyt.) (ratio of “cytoplasmic” cells to interphase cells). Note that for Rpl23A only two siRNAs were used.
(0.24 MB XLS)

Table S4 **Summary of numerical data derived from the Rpl29-GFP screen.** For each siRNA the following information is given: plate number (96-well plate identity), cell number (based Hoechst staining), relative cell number (cell number in the respective siRNA containing well divided by average cell number of all three negative controls of same 96-well plate), GFP-positive cells (interphase cells expressing Rpl29-GFP), hit-classified cells (cells classified as “nuclear” based on restriction of Rpl29-GFP to the cell nucleus), and hit rate (ratio of hit-classified cells to GFP-positive cells). Note that for Rpl23A only two siRNAs were used.
(0.15 MB XLS)

Table S5 **Phenotypic classification of targets with respect to different nuclear 40S biogenesis defects based on nucleolar or nucleoplasmic accumulation of Rps2-YFP.** The average ratio of “nucleoplasmic” to all hit-classified cells (sum of “nucleolar” and “nucleoplasmic” classified cells) for the two siRNAs with the highest hit rates is given for each target in the Rps2-YFP screen. A low value (approaching zero) indicates a nucleolar accumulation of the reporter, whereas a high value (approaching one) indicates a nucleoplasmic accumulation of the reporter. As a measure for the quality of this classification, the difference between the two scoring siRNAs is also given.
(0.02 MB XLS)

Text S1 **Supplemental experimental procedures.**
(0.06 MB DOC)

## Abbreviations

Exp5: Exportin 5
GFP: green fluorescent protein
RNAi: RNA interference
RP: ribosomal proteins
RPL: ribosomal proteins of the large subunit
RPS: ribosomal proteins of the small subunit
SSU processome: small subunit processome
YFP: yellow fluorescent protein
