# Supplementary figures and images for: A Protein Inventory of Human Ribosome Biogenesis Reveals an Essential Function of Exportin 5 in 60S Subunit Export
Source: PLoS Biol. 2010 Oct 26;8(10):e1000522. doi: 10.1371/journal.pbio.1000522 (PMC2964341; doi:10.1371/journal.pbio.1000522)

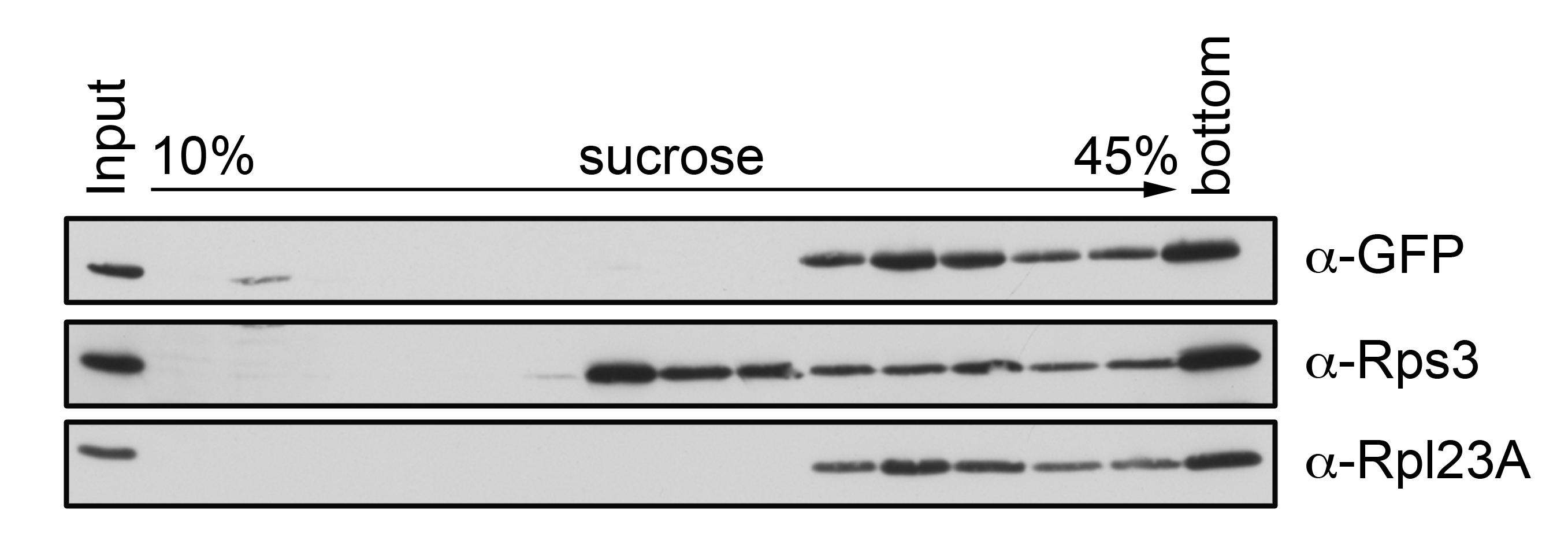

Supplement: Figure S1 — Rpl29-GFP is incorporated into 60S subunits. Extract from HeLa Rpl29-GFP expressing cells (induced with 125 ng/ml tetracycline for 14 h followed by 6 h chase in tetracycline-free medium) was separated on a 10% to 45% sucrose gradient (50 mM Hepes/KOH pH 7.5, 100 mM KCH3CO2, 3 mM MgCl2). Protein in gradient fractions was precipitated and analyzed by immunoblotting using the indicated antibodies. (0.39 MB TIF) [file pbio.1000522.s001.tif]

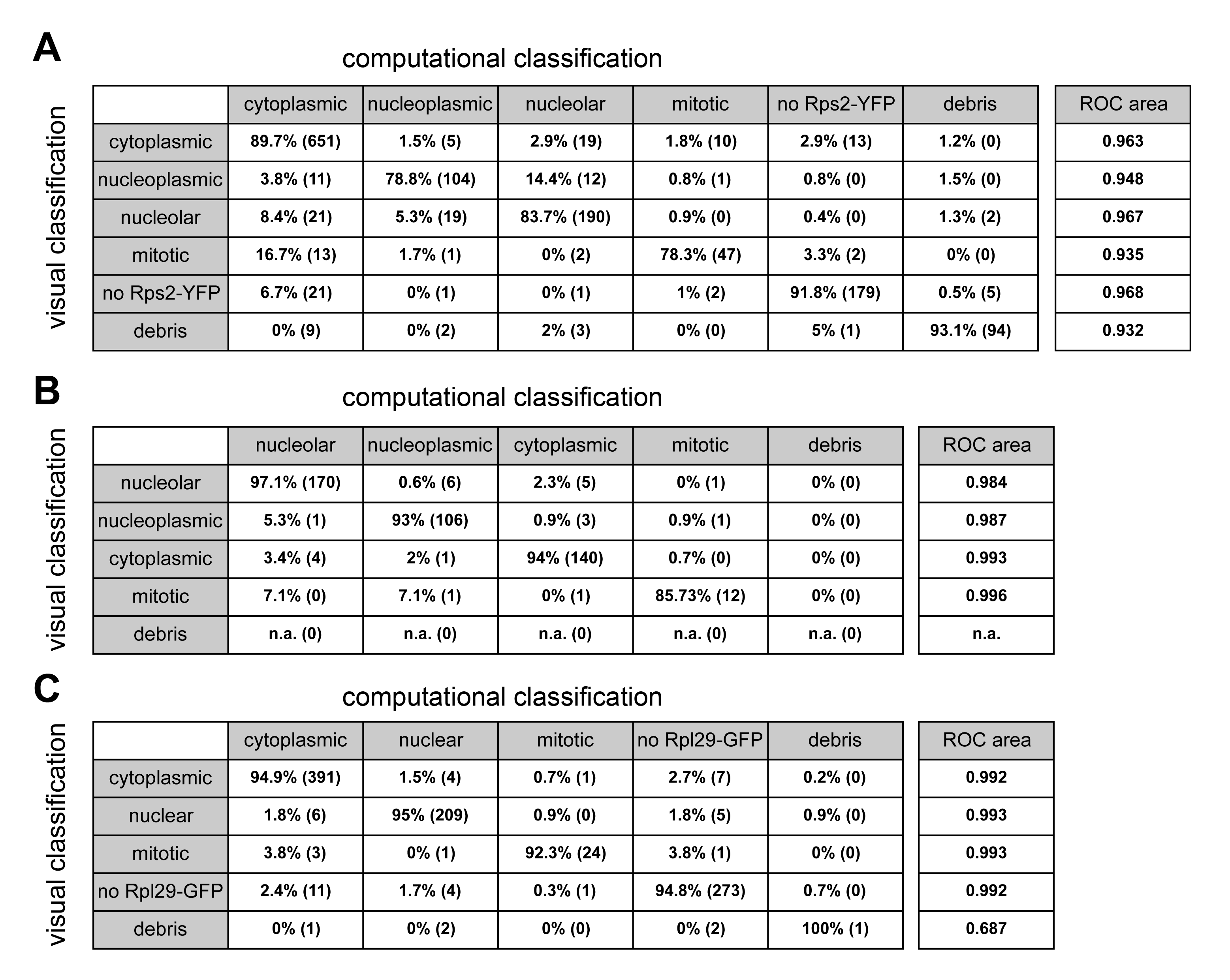

Supplement: Figure S2 — Confusion matrices of phenotypic classification. (A) Cross-validation of visual and computational cell classification for HeLa Rps2-YFP cells. Cells were visually classified into the following predefined phenotypes: cytoplasm (prominent cytoplasmic Rps2-YFP signal), nucleoplasm (predominant nucleoplasmic Rps2-YFP signal), nucleolus (predominant nucleolar Rps2-YFP signal), no Rps2-YFP (cells not expressing Rps2-YFP), mitotic (cells in mitosis), and debris (cell debris; image segmentation errors). Subsequently, computational classification was performed based on the visual classification. The confusion matrix shows the degree of accordance between visual and computational classification. Absolute numbers of classified cells are given in brackets. As a measure for the quality of computational classification, the ROC (receiver operating characteristic) area was calculated. Note that an ROC area >0.9 is considered as excellent. (B) Cross-validation of visual and computational cell classification for cells analyzed by Enp1 IF cells. Predefined phenotypes are: nucleolus (predominantly nucleolar Enp1 IF signal), nucleoplasm (homogenous nuclear Enp1 IF signal), cytoplasm (prominent cytoplasmic Enp1 IF signal), mitotic (cells in mitosis), and debris (cell debris or image segmentation errors). Note that no debris was present in the training set for this readout. (C) Cross-validation of visual and computational cell classification for HeLa Rpl29-GFP cells. Predefined phenotypes are: cytoplasm (nucleolar and cytoplasmic Rpl29-GFP signal), nucleus (nucleolar and nucleoplasmic Rpl29-GFP signal), no Rpl29-GFP (cells not expressing Rpl29-GFP), mitotic (cells in mitosis), and debris (cell debris or image segmentation errors). (0.57 MB TIF) [file pbio.1000522.s002.tif]

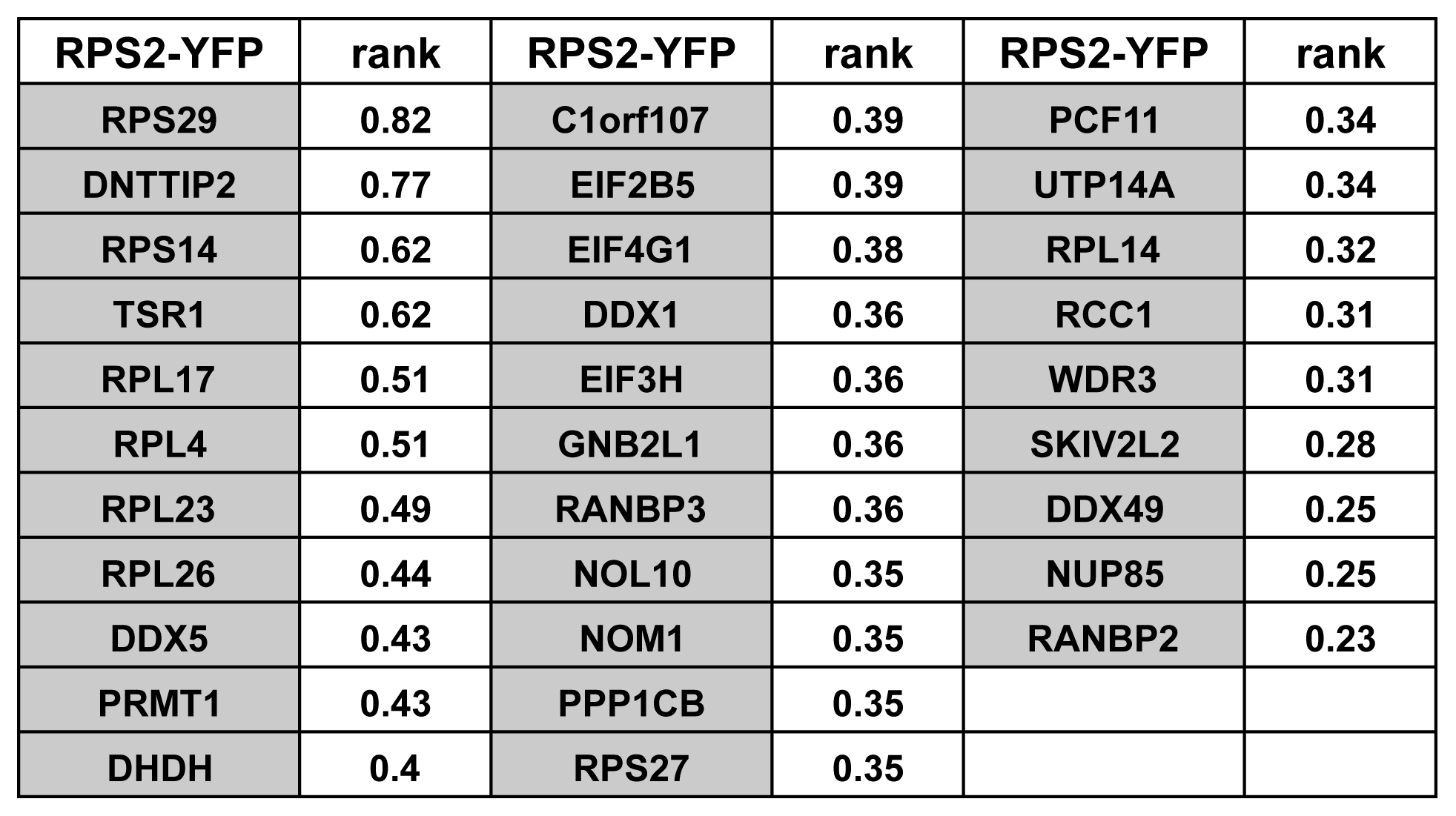

Supplement: Figure S3 — List of additional targets identified as hits at 25 nM siRNA concentration using the Rps2-YFP readout. High confidence hits from Rps2-YFP screen at 25 nM, which were not detected in the screen at 10 nM. Ranking was performed as described. (0.21 MB TIF) [file pbio.1000522.s003.tif]

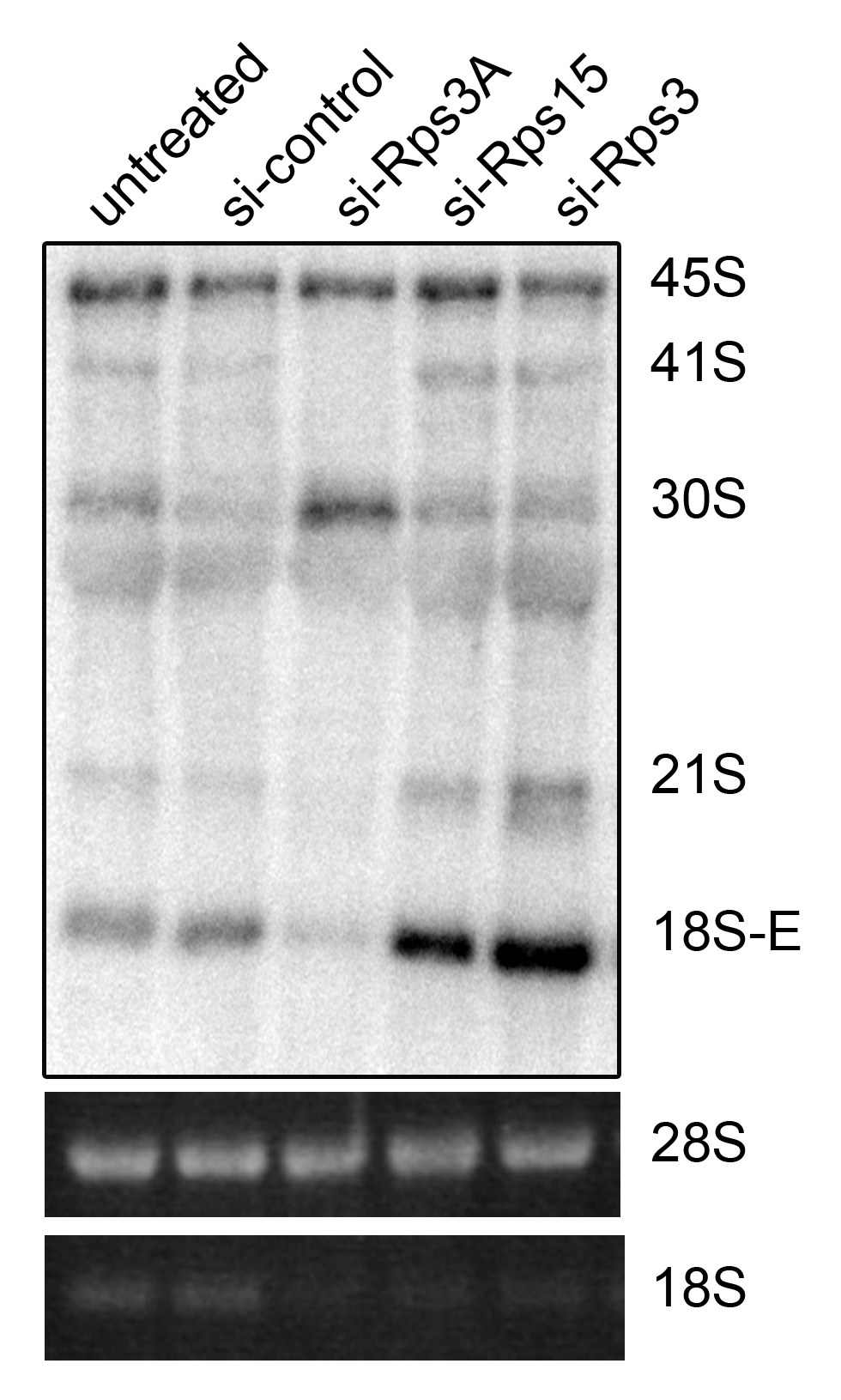

Supplement: Figure S4 — Depletion of different RPS causes distinct rRNA processing defects. RNA isolated from HeLa cells treated with the indicated siRNAs (15 nM) for 72 h was analyzed by Northern blotting using a 5′-ITS1 specific probe [31]. Note that RpsA depletion caused accumulation of a nuclear rRNA species (30S). 18S-E rRNA, which is both nuclear and cytoplasmic, accumulated upon depletion of Rps15 and Rps3. The accumulation of 18S-E rRNA is more pronounced upon depletion of Rps3, a RPS classified to be required for cytoplasmic 40S maturation. (0.78 MB TIF) [file pbio.1000522.s004.tif]

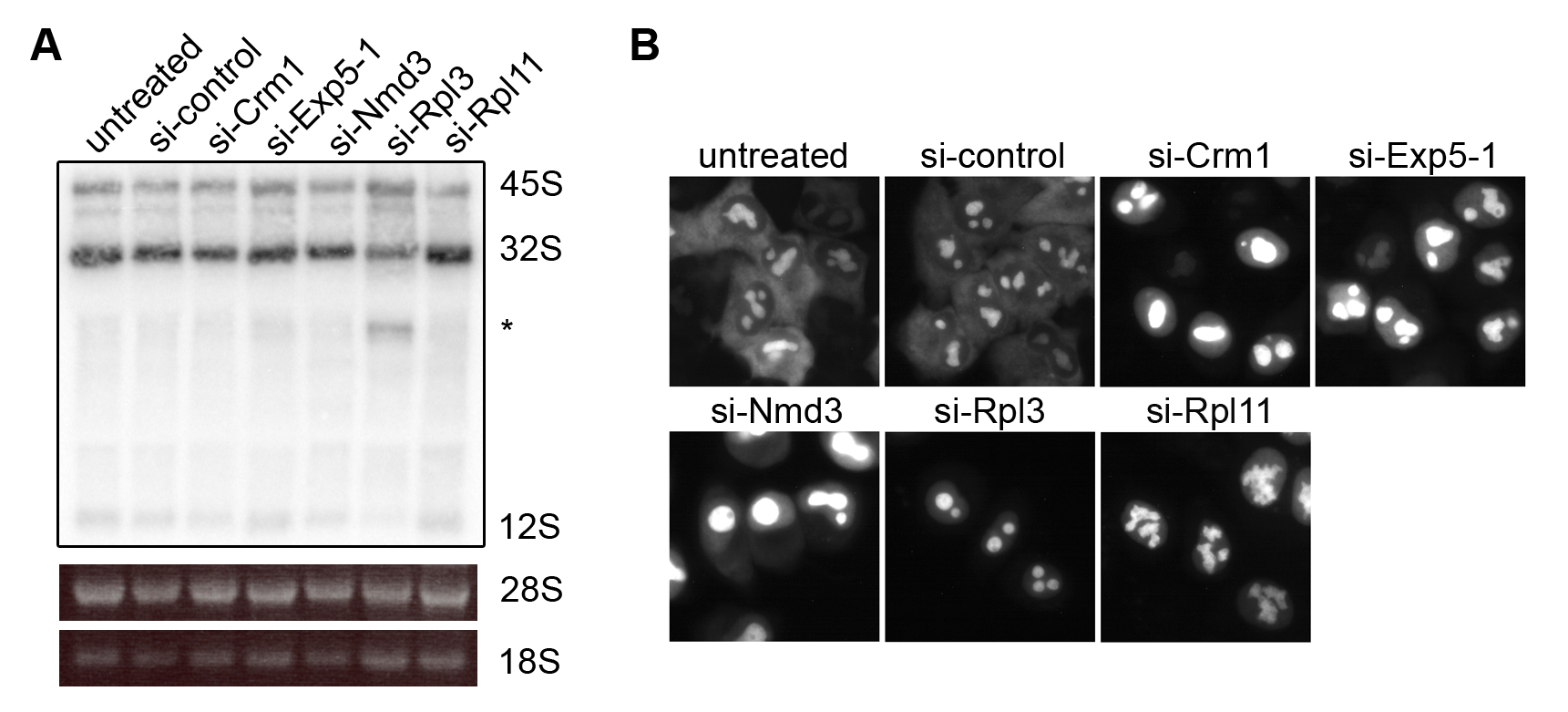

Supplement: Figure S5 — Depletion of Exp5 does not cause rRNA processing defects in the 28S rRNA processing pathway detectable by the ITS2 probe. (A) HeLa Rpl29-GFP cells were treated with the indicated siRNAs (15 nM). After 52 h of RNAi, Rpl29-GFP expression was induced for 14 h by addition of tetracycline, followed by incubation in tetracycline-free medium for 6 h and subsequent RNA extraction. Isolated RNA was analyzed by Northern blotting using an ITS2-d/e [31] specific probe. Ethidium bromide staining of 18S and 28S rRNA is shown as loading control. Note that Exp5 depletion caused no visible rRNA processing defects (similar to Crm1 RNAi), whereas rRNA processing defects are observed upon Rpl3 RNAi (aberrant rRNA species marked with *). Allstars siRNA (Qiagen) was used as a negative control. (B) As a phenotypic readout for efficient RNAi, images of HeLa Rpl29-GFP cells from experiment shown in (A) were taken by epifluorescence microscopy. (0.65 MB TIF) [file pbio.1000522.s005.tif]

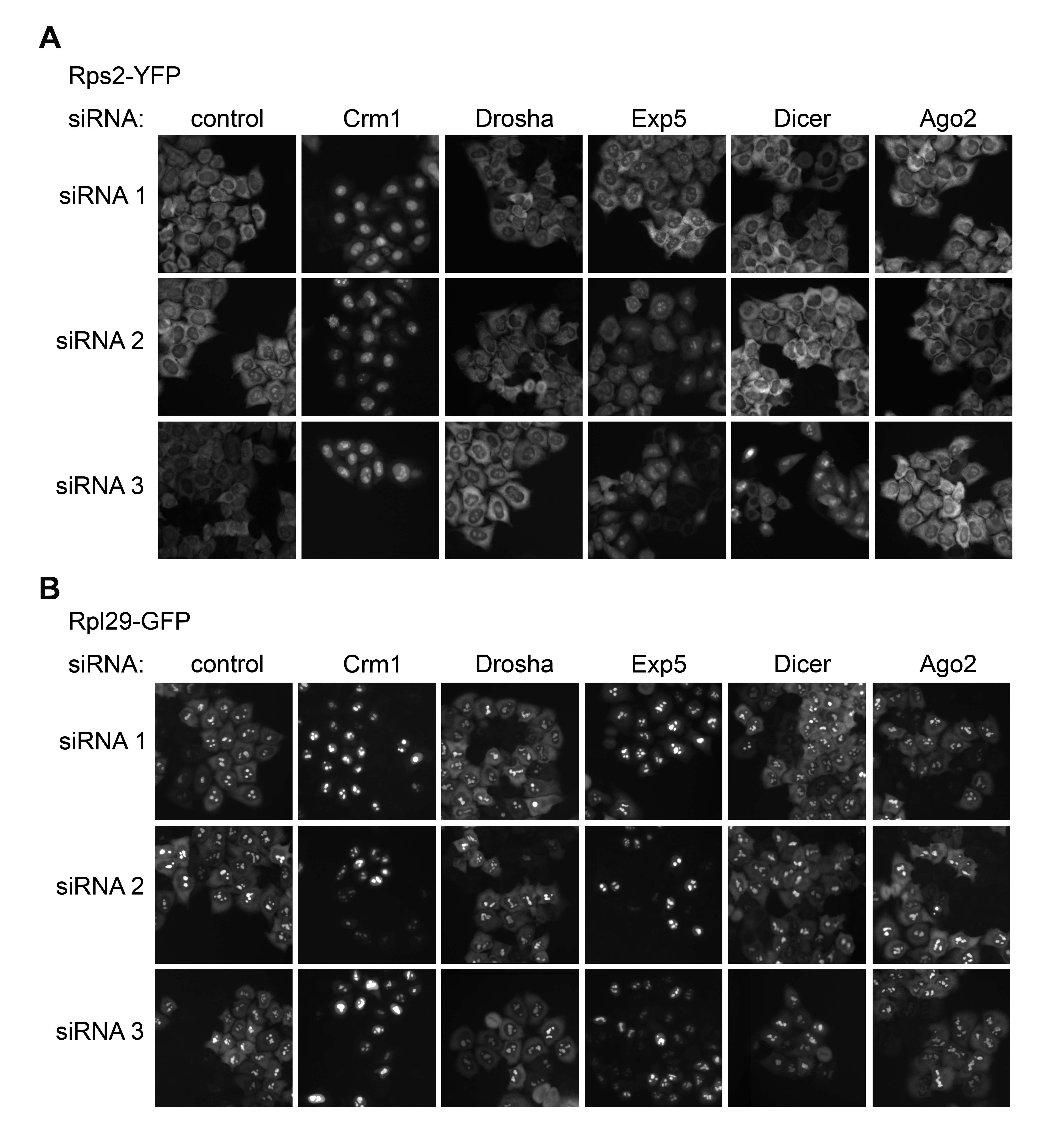

Supplement: Figure S6 — Analysis of 40S and 60S biogenesis defects upon RNAi against miRNA biogenesis factors. (A) HeLa Rps2-YFP cells were treated with the indicated siRNAs (10 nM). RNAi against Crm1 served as positive control, and the Allstars siRNA (Qiagen) was used as a negative control. After 58 h of RNAi, Rps2-YFP expression was induced for 14 h by addition of tetracycline and cells were subsequently fixed. Images were taken by the screening microscope (BD pathway 855). (B) HeLa Rpl29-GFP cells were treated with the indicated siRNAs (10 nM). After 52 h of RNAi, Rpl29-GFP expression was induced for 14 h by addition of tetracycline, followed by incubation in tetracycline-free medium for 6 h and subsequent fixation. Images were taken by the screening microscope (BD pathway 855). Note that for Exp5, only siRNA 2 was also used in the original screening analysis. SiRNA 1 and siRNA 3 are additional, independent siRNAs not used in the original screening and also different from those used in Figure 5. (6.21 MB TIF) [file pbio.1000522.s006.tif]

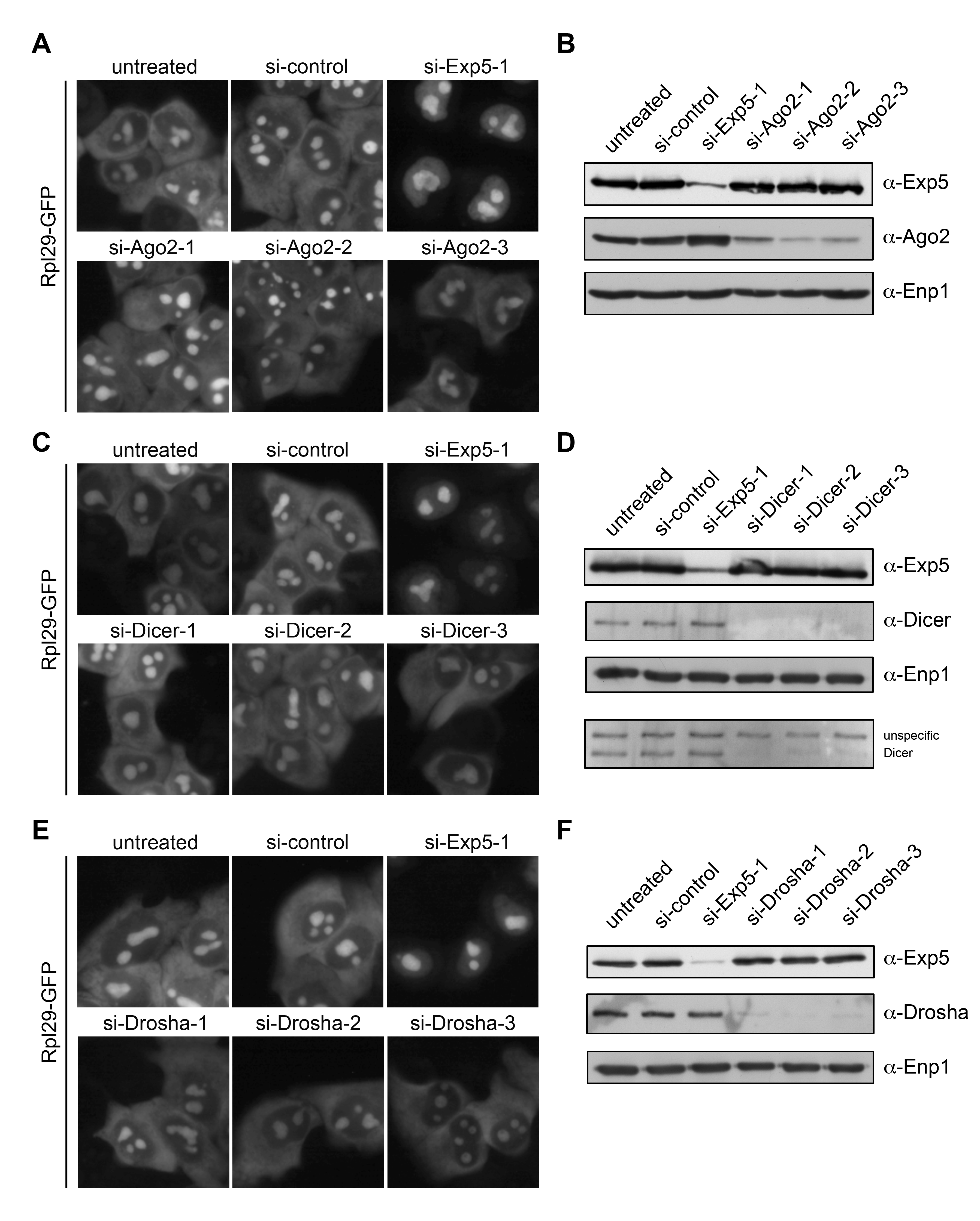

Supplement: Figure S7 — Analysis 60S biogenesis defects after depletion of miRNA biogenesis factors. (A) HeLa Rpl29-GFP cells were treated with the indicated siRNAs (10 nM). After 52 h of RNAi, Rpl29-GFP expression was induced for 14 h by addition of tetracycline, followed by incubation in tetracycline-free medium for 6 h and subsequent fixation. Images were taken by epifluorescence microscopy. (B) Western blot analysis of extracts from cells derived from the experiment shown in (A). Enp1 levels were used as loading control. (C) Experiment as described in (A) with indicated siRNAs. (D) Western blot analysis of extracts from cells derived from the experiment shown in (C). Enp1 levels and a weak unspecific band served as loading controls. (E) Experiment as described in (A) with indicated siRNAs. (F) Western blot analysis of extracts from cells derived from the experiment shown in (A). Enp1 levels were used as loading control. (3.62 MB TIF) [file pbio.1000522.s007.tif]

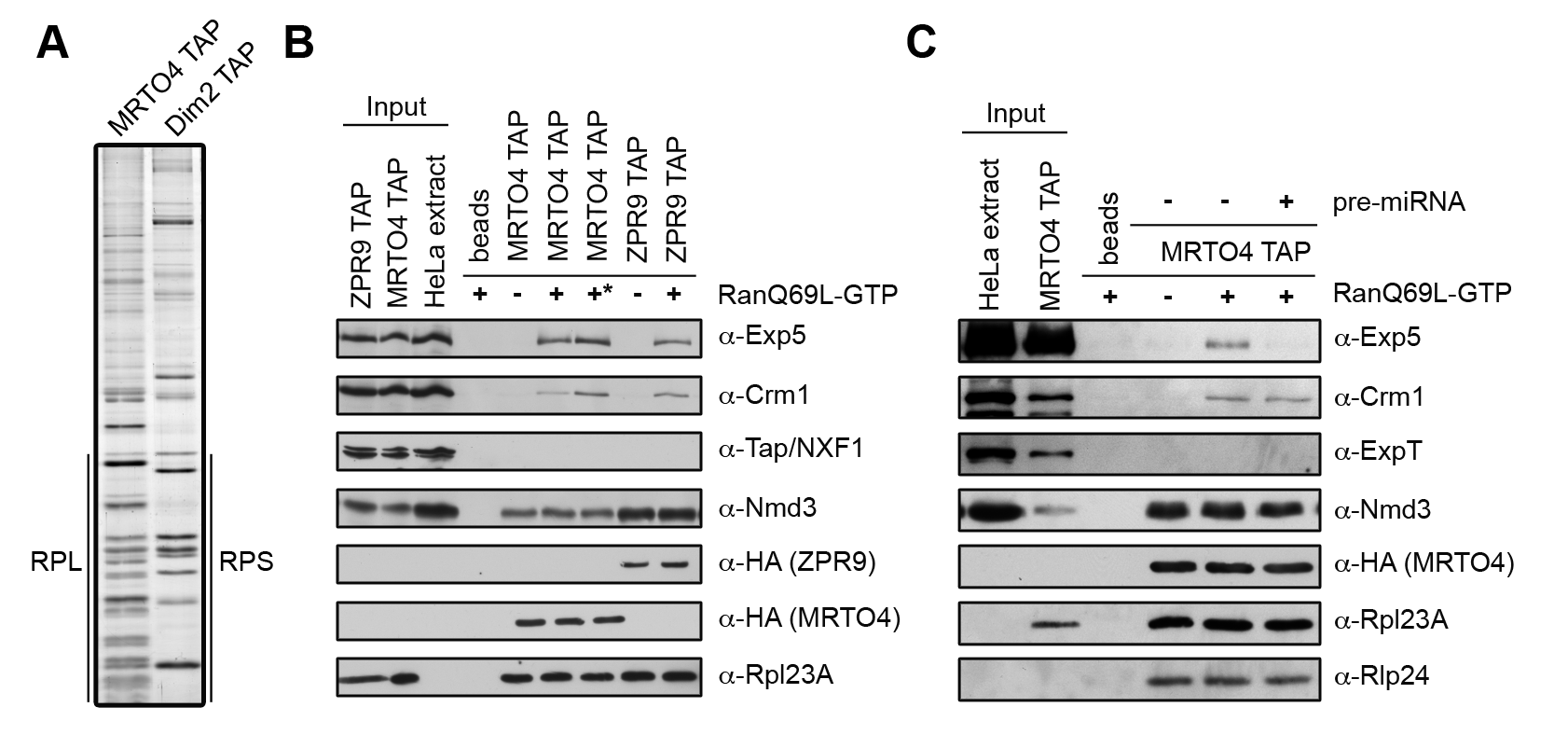

Supplement: Figure S8 — Exportin 5 binding to pre-60S particles isolated by tandem affinity purification. (A) Silver-stained PAA-SDS gel of pre-60S and pre-40S particles isolated on TAP-tagged MRTO4 and Dim2, respectively. Large ribosomal proteins (RPL) and small ribosomal proteins (RPS) isolated on TAP-tagged MRTO4 and Dim2, respectively, are indicated. (B). Exportin 5 binds to pre-60S particles isolated on two different TAP-tagged pre-60S-associated trans-acting factors. Pre-60S particles were purified from HEK293 cells (Input ZPR9-TAP, MTRO4-TAP) bearing either an inducible copy of TAP-tagged MRTO4 or TAP-tagged ZPR9 24 h after tetracycline addition. The purified particles were incubated with tRNA-depleted, postribosomal HeLa cell extract (input HeLa extract) in the absence or presence of 10 µM RanQ69L-GTP. RanQ69LΔC, which cannot be dissociated from its cargo, was used in the sample marked with an asterisk and enhances both Exp5 and Crm1 retrieval. Anti-HA IgG beads were used as a control for unspecific binding of exportins in the presence of RanQ69L-GTP. (C) Pre-miRNA competes for RanGTP-dependent binding of Exportin 5 to pre-60S particles. Experiment was performed essentially as in (B). The pre-miRNA competition experiment was performed by addition of in vitro transcribed pre-miRNA-31 [56] to a final concentration of 10 µM. (0.25 MB TIF) [file pbio.1000522.s008.tif]
