# Supplementary material for: A Protein Inventory of Human Ribosome Biogenesis Reveals an Essential Function of Exportin 5 in 60S Subunit Export
Source: PLoS Biol. 2010 Oct 26;8(10):e1000522. doi: 10.1371/journal.pbio.1000522 (PMC2964341; doi:10.1371/journal.pbio.1000522)
Supplement: Text S1 — Supplemental experimental procedures. (0.06 MB DOC) [file pbio.1000522.s014.doc]

**Text S1**

**Supplemental experimental procedures**

**Image and data analysis**

The data analysis was performed in three major steps. First, image processing was used to identify single cells and extract their properties. Second, each single cell was classified into one of several phenotypic classes using supervised machine learning. Third, the dataset was analyzed and processed (hit definition) to generate lists of targets that cause defects in ribosomal biogenesis when depleted.

*Image Processing*

To identify cells and extract their properties, the CellProfiler [1 program was used with custom modifications. First, image intensities were rescaled. Then, cell nuclei were identified using Otsu thresholding and Watershed algorithm to separate those nuclei, which were too close together and not identified as single nuclei during thresholding. Next, a cytoplasmic area was selected as a ring around the cell nucleus (width of 12 pixels). Using the detected objects (nucleus and cytoplasm) as masks, we extracted cell features such as intensity mean and standard deviation values, textural properties, and morphological descriptors.

*Single cell-based supervised classification*

We classified cells into predefined phenotypes (Figure 1, Figure S2). For HeLa Rps2-YFP and Enp1 IF, we distinguished cells with prominent signal in the nucleolus (‘nucleolar’), nucleoplasm (‘nucleoplasmic’) or cytoplasm (‘cytoplasmic’). HeLa Rpl29-GFP cells were divided in classes showing an exclusively nuclear signal (‘nuclear’) or a nucleolar and cytoplasmic signal (‘cytoplasmic’). Additionally, we defined classes of cells not expressing Rps2-YFP (‘no Rps2-YFP’) or Rpl29-GFP (‘no Rpl29-GFP’) as well as classes of mitotic cells (‘mitotic’) and of cell debris (‘debris’). From the acquired data, we concluded that multi-parametric, nonlinear analysis outperforms simpler methods. We developed a program, the Advanced Cell Classifier (http://acc.ethz.ch), which allows for easy labelling of cells, for choosing between different classification methods, and that performs the analysis on the entire data set. We tried the performance of more than 20 available supervised classification methods. The best performance was achieved by the neural network method [2. The confusion matrices of cross validation for the three data sets are depicted in Figure S2. We observe a very high accuracy of phenotype assignment, i.e. a high accordance of visual and computational classification.

*Data analysis and hit definition*

Based on the results of supervised classification, we calculated a ‘hit rate’ for each individual siRNA by taking the ratio of cells displaying a ribosome biogenesis phenotype to all biologically relevant cells (i.e. all interphase cells that express the Rsp2-YFP or the Rpl29-GFP reporter). Note that those siRNAs having a strong effect on cell survival (less than 50 reporter-positive cells per well, compared to an average of 490 cells for Rps2-YFP and 290 for Rpl29-GFP) were excluded from subsequent analysis. This led to an exclusion of 4 siRNAs for the Rps2-YFP analysis at 10 nM, 16 siRNAs for the Rps2-YFP analysis at 25 nM, 1 siRNA for Enp1 IF analysis and 17 siRNAs for the Rpl29-GFP analysis.

Next, a two-step filter was applied for the generation of high confidence hit lists. First, all siRNAs leading to at least twice the hit rate of the average hit rate of negative controls (AllStars, Qiagen) present on the same 96-well plate were selected. From the three negative controls (AllStars, Qiagen), the one with the lowest hit rate was excluded from this calculation. The exclusion of the negative control with the lowest hit rate increases stringency in this step. Targets represented by at least two siRNAs that produced a phenotype after this first filtering were considered ‘low confidence’ hits.

To compare low confidence hits from different 96-well plates, we calculated a performance index for each siRNA according to the positive (Crm1 RNAi) and negative controls (AllStars, Qiagen) of the respective 96-well plate. For this, the average hit rate of the two negative controls with the highest hit rate was subtracted from the hit rate of a siRNA of interest. This value was then divided by the difference of the average hit rate of the two best positive controls (positive controls with highest hit rates) and the average hit rate of the two worst negative controls (negative controls with highest hit rate). Selection of the two positive controls with highest hit rate increases stringency, as it prevents high ranking of a target gene in case one inefficient positive control was present on the respective plate. To rank all different targets from different 96-well plates relative to each other, the average of the resulting indices for two siRNAs was used. For targets that had been assigned as hits through three siRNAs, the two siRNAs giving the highest rank were used for rank calculation. Note that a target ranking procedure could not be performed for cytoplasmic Enp1 hits due to the lack of an appropriate positive control for this phenotype on the screening plates.

Second, to obtain a compilation of high confidence hits, a rank cutoff was defined for the Rps2-YFP readout as 0.2. With this, the lowest high confidence hit at 10 nM siRNA concentration, TBL3, displayed an average hit rate more than 3 times higher than the negative controls from the same plate. To set the rank cutoff for the Rpl29-GFP and Enp1 IF readout accordingly, the overall separation of positive and negative controls for the used read-outs was calculated. For this, the average hit rate of negative controls was subtracted from the average hit rate of positive controls for each 96-well plate. Then, an average for all 96-well plates was calculated as the overall separation of positive and negative controls achieved with the respective readout. For Rps2-YFP a value of 0.84, for Enp1 IF a value of 0.54 and for Rpl29-GFP a value of 0.69 was calculated. The rank cutoff for high confidence nuclear Enp1 IF hits was then calculated as 0.2x(0.84/0.54)=0.31 and set to 0.3. The rank cutoff for high confidence Rpl29-GFP hits was calculated as 0.2x(0.84/0.69)=0.24 and set to 0.25. For cytoplasmic Enp1 IF, the high confidence hit cutoff was set to 0.25. With this, the lowest high confidence hit, Rps26, displayed an average hit rate more than 3 times as high as the average hit rate of negative controls on the respective plate.

**Protein purification**

The purification of RanQ69L-GTP and RanQ69LΔC [3 has been described.

**Antibodies**

Recombinant His-tagged GFP and human Rps3A were used as antigens in rabbits to generate α-GFP and α-Rps3A antibodies, respectively. The antibody against TAP/NXF1 was raised against the peptide C-QQALDLKGLRSDPDLVAQ. All antibodies were affinity-purified on their respective antigens. The antibody against Drosha is commercially available (Abcam; ab12286), the Ago2 and Dicer antibodies have been published 4,5.

**RNAi**

RNAi analysis of miRNA biogenesis components was performed as described in the Materials and Methods. For RNA extraction and subsequent Northern analysis, RNAi was performed on HeLa cells using the described protocol, but adjusted from 96-well format to 6-well format. Sense strands of the used siRNAs are: Rps3 (5’-GCAAGAUGGCAGUGCAAAU), Rps3A (5’-GCUCACGUUGAUGUCAAG), Rps15 (5’-UCACCUACAAGCCCGUAAA), Rpl3 (5’-GCAAGCUGAUCAAGAACAA), Rpl11 (5’-GGGAGUAUGAGUUAAGAAA), Ago2-1 (5’-ACGGGUCUGUGGUGAUAAA), Ago2-2 (5’-AGAUCCAUACGUCCGUGAA), Ago2-3 (5’-GCACCGGCAGGAGAUCAUA), Dicer-1 (5’-CGAUCCUAUGUUCAAUCUA), Dicer-2 (5’-UGUGCUAUCUGGAUCCUAG), Dicer-3 (5’-CACUGAUUCUGCAUAUGAA), Drosha-1 (5’-GAAGCUCGAUGAAGAUUUA), Drosha-2 (5’-GGGAUUAACACCUUGAUAA), Drosha-3 (5’-CGAUCAACUGGAUCGUGAA).

**Supplemental references**

1. Carpenter AE, Jones TR, Lamprecht MR, Clarke C, Kang IH, et al. (2006) CellProfiler: image analysis software for identifying and quantifying cell phenotypes. Genome Biol 7: R100.

2. Bishop C (1996) Neural Networks for Pattern Recognition. Oxford University Press.

3. Kutay U, Bischoff FR, Kostka S, Kraft R, Gorlich D (1997) Export of importin alpha from the nucleus is mediated by a specific nuclear transport factor. Cell 90: 1061-1071.

4. Rudel S, Flatley A, Weinmann L, Kremmer E, Meister G (2008) A multifunctional human Argonaute2-specific monoclonal antibody. RNA 14: 1244-1253.

5. Kotaja N, Bhattacharyya SN, Jaskiewicz L, Kimmins S, Parvinen M, et al. (2006) The chromatoid body of male germ cells: similarity with processing bodies and presence of Dicer and microRNA pathway components. Proc Natl Acad Sci U S A 103: 2647-2652.
